# Ethanol-Producing *Enterocloster bolteae* Is Enriched in Chronic Hepatitis B-Associated Gut Dysbiosis: A Case–Control Culturomics Study

**DOI:** 10.3390/microorganisms11102437

**Published:** 2023-09-28

**Authors:** Reham Magdy Wasfy, Babacar Mbaye, Patrick Borentain, Maryam Tidjani Alou, Maria Leticia Murillo Ruiz, Aurelia Caputo, Claudia Andrieu, Nicholas Armstrong, Matthieu Million, Rene Gerolami

**Affiliations:** 1 IHU Méditerranée Infection, 13005 Marseille, Francetidjani_maryam@hotmail.com (M.T.A.); claudia.andrieu1@ap-hm.fr (C.A.);; 2 MEPHI, IRD, Aix-Marseille Université, 13005 Marseille, France; 3 Unité Hépatologie, Hôpital de la Timone, APHM, 13005 Marseille, France; patrick.borentain@ap-hm.fr; 4 Assistance Publique-Hôpitaux de Marseille (APHM), 13005 Marseille, France

**Keywords:** dysbiosis, gut microbiota, chronic hepatitis B virus infection, *Enterocloster bolteae*, culturomics, metagenomics

## Abstract

Background: Hepatitis B virus (HBV) infection is a global health epidemic that causes fatal complications, leading to liver cirrhosis and hepatocellular carcinoma. The link between HBV-related dysbiosis and specific bacterial taxa is still under investigation. *Enterocloster* is emerging as a new genus (formerly *Clostridium*), including *Enterocloster bolteae*, a gut pathogen previously associated with dysbiosis and human diseases such as autism, multiple sclerosis, and inflammatory bowel diseases. Its role in liver diseases, especially HBV infection, is not reported. Methods: The fecal samples of eight patients with chronic HBV infection and ten healthy individuals were analyzed using the high-throughput culturomics approach and compared to 16S rRNA sequencing. Quantification of ethanol, known for its damaging effect on the liver, produced from bacterial strains enriched in chronic HBV was carried out by gas chromatography–mass spectrometry. Results: Using culturomics, 29,120 isolated colonies were analyzed by Matrix-Assisted Laser Desorption/Ionization Mass Spectrometry (MALDI–TOF); 340 species were identified (240 species in chronic HBV samples, 254 species in control samples) belonging to 169 genera and 6 phyla. In the chronic HBV group, 65 species were already known in the literature; 48 were associated with humans but had not been previously found in the gut, and 17 had never been associated with humans previously. Six species were newly isolated in our study. By comparing bacterial species frequency, three bacterial genera were serendipitously found with significantly enriched bacterial diversity in patients with chronic HBV: *Enterocloster*, *Clostridium*, and *Streptococcus* (*p* = 0.0016, *p* = 0.041, *p* = 0.053, respectively). However, metagenomics could not identify this enrichment, possibly concerning its insufficient taxonomical resolution (equivocal assignment of operational taxonomic units). At the species level, the significantly enriched species in the chronic HBV group almost all belonged to class *Clostridia*, such as *Clostridium perfringens*, *Clostridium sporogenes*, *Enterocloster aldenensis*, *Enterocloster bolteae*, *Enterocloster clostridioformis*, and *Clostridium innocuum.* Two *E. bolteae* strains, isolated from two patients with chronic HBV infection, showed high ethanol production (27 and 200 mM). Conclusions: Culturomics allowed us to identify *Enterocloster* species, specifically, *E. bolteae*, enriched in the gut microbiota of patients with chronic HBV. These species had never been isolated in chronic HBV infection before. Moreover, ethanol production by *E. bolteae* strains isolated from the chronic HBV group could contribute to liver disease progression. Additionally, culturomics might be critical for better elucidating the relationship between dysbiosis and chronic HBV infection in the future.

## 1. Introduction

Hepatitis B virus (HBV) infection is a global health burden. Around 257 million people are HBV-positive worldwide [1]. HBV infection constitutes the primary driver of severe complications such as cirrhosis and hepatocellular carcinoma (HCC) [2]. Antiviral treatment has proven to slow HBV progression; however, the disease occurs in a small percentage of people even when a viral load is undetectable (50 IU/mL) [3]. Therefore, the pathophysiologic mechanisms underlying the evolution of chronic infection with HBV are still incompletely understood and deserve further investigation.

Gut microbiota has a complicated and mutually beneficial relationship with the host and plays a vital role in the host’s metabolism, nutrition, pathological processes, and immune function [4]. The link between gut microbiota, its derivatives, and the pathophysiology of the liver has attracted considerable interest [5,6]. The liver receives various gut-derived substances (bacterial products, environmental toxins, and food antigens) via the biliary tract, portal vein, and systemic circulation due to the structural link to the intestine known as the gut–liver axis [7]. Bacteria and bacterial products from the gut microflora have been associated with systemic inflammation and severe liver diseases [8,9]. One of those products is ethanol, which is known to damage liver sinusoidal endothelial cells and induces centrilobular sinusoidal collapse, which reduces blood flow and impairs the microcirculatory exchange of nutrients and clearance of waste products [10].

In fact, different studies reported significant changes in gut microbiota composition in patients with chronic HBV infection based on metagenomics analysis, characterized by high abundance in *Proteobacteria* phylum [11] and genera such as *Streptococcus* [12], *Prevotella* [13], *Ruminococcus*, and *Veillonella* [14,15] and low abundance of *Clostridium* [11,16,17]. However, no study has investigated gut microbiota in chronic HBV infection based on cultivation techniques. The high-throughput culturomics approach has been documented in studying gut microbiota [18]. Culturomics provides viable pure cultures, unlike molecular approaches that only give information on the genus level (species-level assignment is uncertain for polyphyletic groups such as *Enterobacteria*), without any information regarding their viability status. In addition, microbial culture using selective media has made it possible to detect and cultivate minority bacterial populations that may pass undetected by genomic technologies [19]. By achieving this, culturomics could eliminate different limitations of metagenomics, such as extraction and bioinformatic bias, yielding difficult discrimination between species owing to short amplicon length [19]. Metagenome-assembled genomes (MAGs) [20] are a new trend that is aimed at overcoming these limitations. However, MAGs are artificial because they represent a bio-informatic reconstruction and may be biased by chimeric and repeat contigs [21]. Moreover, to date, culturomics has enabled the isolation of more than 300 new bacterial species in the human gut that were previously believed to be uncultured by traditional culture methods [18,19].

A previous study has reported the value of culturomics, performed in a case–control study, in defining the missing repertoire of probiotics and beneficial species that could be used in fecal microbiota transplantation (FMT), which requires viable isolated strains [22]. Interestingly, patients with chronic HBV infection who underwent FMT have achieved hepatitis B e-antigen (HBeAg) clearance in different studies [23,24,25]. Additionally, probiotics were reported to function by preventing liver inflammation from impeding the advancement of HCC via antioxidant and anti-metastatic activities [26]. In addition, the role of probiotics, particularly in chronic HBV-related complications such as hepatic encephalopathy, has been established in different studies [27,28,29,30]. As a result, it is critical to distinguish between culturomics, which specifically detects live organisms, and metagenomics, which yields DNA sequences most likely derived from already-dead species and cannot be isolated or multiplied [31]. To date, not all bacteria can grow, even in culturomics [31].

Moreover, among the genus *Clostridium*, a new genus of anaerobic bacteria, namely *Enterocloster*, has emerged as a reclassification due to advances in phylogenetics [32]. *Enterocloster bolteae* (formerly known as *Clostridium bolteae*) is an obligately anaerobic, Gram-positive, rod-shaped, and spore-forming gastrointestinal pathogenic bacterium [33]. *E. bolteae* was reported to produce butyrate, propionate, and acetate, which alter the motility and contraction rate of the gastrointestinal tract attributed to chronic diarrheal episodes [34]. *E. bolteae* appears to produce *beta*-lactamases with resistance to β-lactam antibiotics such as penicillin G, ticarcilllin, and piperacillin–tazobactam [35]. It is known to be present in the stools of most children, with a significantly higher abundance in autistic children [36]. *E. bolteae* showed high abundance in patients with fatty liver disease [37]; however, its role or abundance profile has not been reported in chronic HBV infection. *E. bolteae* was reported to excrete metabolites that are thought to act as neurotoxins [38] and is known to produce ethanol [39]. In patients with chronic HBV infection, alcohol intake is associated with more severe liver disease [40]. Hence, reporting *E. bolteae* in chronic HBV infection is an interesting aspect of our study.

In this small study, we are the first to describe a culturomics-based microbiome profile to the species level in patients with chronic HBV infection and healthy individuals. The species identified by both culturomics and metagenomics for each group of samples were compared, highlighting the complementarity of these two approaches. Our study aims to detect possible endogenous ethanol production by specific bacterial strains enriched in patients with chronic HBV infection.

## 2. Material and Methods

### 2.1. Study Design

A case–control study was carried out in the Hepatology Department of Marseille University Hospital (south-eastern France), Marseille, France, according to STROBE statement guidelines [41] from January to June 2022. The HEPATGUT study was approved by the local ethics committee of l’Institut Hospitalo-Universitaire Méditerranée Infection, Marseille, France (IHUMI, 2020-004), approved by the Protection of Persons Committee (Approval No. CPP: 21.04391.000046—21075), and carried out according to the 2013 Declaration of Helsinki (1975; World Medical Association, 2013) [42]. Informed consent was obtained from the study subjects before their enrolment.

### 2.2. Study Population

The HEPATGUT study included 48 people with HBV and controls, intending to analyze 10 HBV and 10 control samples using culturomics and metagenomics. However, only eight HBV and ten controls have been studied and are reported in the present article after the exclusion of two HBV samples due to misclassification, in order to ascertain perfectly defined cases and controls. The HBV patients were recruited from Marseilles University Hospital. Healthy controls without chronic diseases or on regular medications were recruited using a snowball approach [43]. Data on age, gender, weight, height, and dietary habits were collected. Patients with chronic HBV infection were confirmed to be positive for hepatitis B surface antigen for at least six months; the diagnosis was made according to European Association for the Study of the Liver (EASL) guidelines [44].

Alcoholism and probiotics/antibiotics intake in the previous month were exclusion criteria for both cases and controls. In addition, vegetarians (those who eat only vegetables, fruits, grains, nuts, and occasionally eggs or dairy products) were excluded, as they represent a minority, and this dietary habit has been associated with a distinct gut microbiota [45]. The other exclusion criteria for all patients were as follows: patients with viral hepatitis other than HBV, solid organ transplantation, smoking, immunosuppressive drugs treatment within six months, acute or chronic infectious diseases, other liver disease, metabolic disease, malignancy, and autoimmune diseases. The fecal samples from all participants were collected and kept in sterile plastic tubes containing 1000 µL transport medium; they were immediately (within 15 min) stored at −80 °C until used. The transport medium consisted of 3 g/L NaCl, 0.2 g/L KCL, 0.1 g/L CaCl_2_, 0.1 g/L MgCl_2_, 0.2 g/L KH_2_PO_4_, 0.25 g/L Na_2_S, 0.1 g/L glutathione, and 0.1 g/L uric acid. The pH was adjusted to 7.5, and the medium was filtered at 0.2 µm.

### 2.3. High-Throughput Culturomics Approach

The stool samples (eight samples from patients with chronic HBV infection and ten samples from controls) were cultured according to the culturomics approach previously established in our laboratory [46]. Fast culturomics was applied using four culture conditions, as comprehensively detailed in a previous publication by Naud et al. [47]. Sampling of specimens was carried out in anaerobic conditions. For this purpose, the stool sample was placed in a half-open jar, itself placed under anaerobic conditions in a zip bag (Oxoid, Dardilly, France) complemented by an anaerobic GasPak (Becton Dickinson, Le Pont de Claix, France). The stool samples were aliquoted and frozen at −80 °C until use. One gram of stool sample was diluted in one millileter of phosphate-buffered saline (PBS) (Thermo Fisher Scientific, Illkirch, France), and the diluted samples were directly inoculated on agar plates and also introduced with a syringe for pre-incubation into aerobic and anaerobic blood culture bottles. For direct inoculation, the stool sample was carried out on 5% sheep blood enriched Columbia (COS) agar (BioMérieux, Craponne, France) and inhouse YCFA (yeast extract, casein hydrolysate, fatty acids) agar. The sample was also pre-incubated in two liquid culture media at 37 °C: the commercially available Biomerieux medium and the YCFA medium, supplemented with 2 mL defibrinated sterile sheep blood (BioMerieux, Marcy l’Etoile, France), and 2 mL active rumen filtered at 0.22 µm. Then, the blood culture vials and the directly inoculated agar plates were incubated at 37 °C.

Inoculation on blood agar of the preincubated samples in blood culture bottles was performed for one month on different incubation days: Day 1, Day 3, Day 7, Day 10, Day 15, Day 21, and Day 30 [48]. To this end, the preincubated stool samples were diluted from 10^−1^ to 10^−10^ in PBS to dilute the sample and identify the maximum number of bacterial species. Then, 50 µL of diluted sample was deposed and homogenized onto COS, but only the YCFA + rumen + blood culture condition was also deposited onto YCFA agar. Each agar plate was incubated for 24 h for aerobic conditions or 48 h for anaerobic conditions at 37 °C, mimicked with a zip bag containing an anaerobic GasPak. Bacterial colonies different in appearance, size, or color were subcultured on COS agar. These agars were then incubated according to the culture condition previously described (direct inoculation, aerobic bottle + rumen + blood, anaerobic bottle + rumen + blood, and anaerobic bottle + YCFA + rumen + blood).

The purified resulting colonies were then identified using Matrix-Assisted Laser Desorption/Ionization Mass Spectrometry (MALDI–TOF/MS) according to the manufacturer’s instructions [49,50]. Each colony was deposited in duplicate on a 96 MSP microplate and covered with 2 μL matrix solution. The solution was made of saturated α-cyano-4-hydroxycinnamic acid, 50% acetonitrile, and 2.5% trifluoroacetic acid. Measurements were performed with a MicroFlex LT/SH (Bruker, Bremen, Germany). Spectra were recorded in the positive linear mode. Data were automatically acquired using FlexControl v.3.4 and MALDI Biotyper Compass v4.1 software for the run preparation and bio-typing analysis. The spectra of different species were compared to MBT Compass BDAL Library v.11 (Bruker) containing 10,833 references of spectra (dispatched between 3893 species), as well as our laboratory database Culturomics containing 9973 references of spectra (dispatched between 2186 bacterial species) (accessed in October 2022). A score > 1.9 allowed identification at the species level. In the case of unidentified colonies by MALDI–TOF (score < 1.9), DNA from the unidentified colonies was extracted, amplified, purified, and analyzed, as described in [22]. The sequences with a similarity percentage under 98.65 or 95% [51] were identified as new species or new genera, respectively, and described according to the taxonogenomics concept [52]. If the bacterial species identification was not interpretable, 16S ribosomal DNA gene sequencing was performed.

The isolated species were compared to a recently published cultured human bacteria repertoire describing the bacterial species that have been cultured at least once from humans [53].

### 2.4. Method of 16S Ribosomal DNA Gene Amplification and Sequencing

A mechanical treatment was first performed on samples with powder glass beads acid washed (G4649-500 g Sigma, St. Louis, MO, USA) and 0.5 mm glass beads cell disruption media (Scientific Industries, Bohemia, NY, USA) using a FastPrep-24™ 5G Grinder (mpBio) at maximum speed (6.5 m/s) for 90 s. Then, the samples were treated through two kinds of lysis methods, namely, method 1 and method 5, according to a previous study [54]. Method 1 is associated with classical lysis and protease step, followed by purification with E.Z.N.A Tissue DNA Kit (Omega bio-tek, Norcross, GA, USA), while method 5 is associated with using a deglycosylation step and purification on the EZ1 Advanced XL device with the EZ1 Qiagen tissue kit (Qiagen, Courtaboeuf, France). The libraries were constructed according to the protocol: “16S Metagenomic Sequencing Library Preparation” (Illumina Inc, San Diego, CA, USA). For each extraction protocol, the 16S “V3-V4” region of DNA samples was amplified by a PCR of 45 cycles using the Kapa HiFi Hotstart ReadyMix 2x (Roche, Basel, Switzerland). The primers used contain a conserved V3-V4 region with overhang Illumina adapters (FwOvAd_341F: 5′TCGTCGGCAGCGTCAGATGTGTATAAGAGACAGCCTACGGGNGGCWGCAG3′; RevOvAd_785R: 5′GTCTCGTGGGCTCGGAGATGTGTATAAGAGACAGGACTACHVGGGTATCTAATCC3′). The same probes used to extract the samples were used as a negative control in addition to a blank of PCR containing the water used to prepare the mix. After purification on AMPure beads (Beckman Coulter Inc, Fullerton, CA, USA), concentration was measured using high-sensitivity Qubit technology (Beckman Coulter Inc., Fullerton, CA, USA), and dilution to 3.5 ng/µL was performed. At this step, the library of method 1 was pooled, volume to volume, to the library of method 5, and Illumina sequencing adapters and dual-index barcodes were added to the amplicon. After purification on AMPure beads (Beckman Coulter, Brea, CA, USA), this library was pooled with other multiplexed samples. The concentration of the pooled libraries was quantified by a Qubit assay with the high sensitivity kit (Life Technologies, Carlsbad, CA, USA) and diluted to 4 nM. Before loading for sequencing on MiSeq (Illumina, San Diego, CA, USA), the pool was denatured and diluted at 8 pM. Automated cluster generation and paired-end sequencing with dual-index reads were performed in a single 39 h run in a 2 × 250 bp. The paired reads were filtered according to the read qualities. The raw data were configured in fastq files for R1 and R2 reads.

### 2.5. Measurement of Ethanol Production by Strains Enriched in Chronic HBV Samples

The bacteria were grown in a yeast–peptone–glucose (YPG) medium composed of the following components: 10 g yeast extract (BioMérieux, Craponne, France), 20 g peptone (BD Diagnostic, Le Pont de Claix, France), 20 g D-glucose (BD Diagnostic, Le Pont de Claix, France), and 1 L of distilled water. Once all ingredients were thoroughly dissolved, the broth was filtered using the RapidFlow™ filtration system (Thermo Fisher, Illkirch, France) with a 0.22 µm membrane, and 10 mL of filtered broth was poured into Hungate tubes. Each tube was inoculated at a concentration of one McFarland to standardize bacterial concentration. Several colonies were picked and suspended in tubes with sterile 0.85% NaCl medium (BioMérieux, Craponne, France), and turbidity was measured using a McFarland densitometer (BioMerieux, Marcy l’Etoile, France).

The strains were grown in three different experiments, with some variations in the protocol in terms of inoculated bacterial concentration and degassing time. Experiment 1: the bacterial suspension was inoculated at one McFarland, and the tubes were degassed for two minutes. Experiment 2: To promote the growth of *E. bolteae* strains, the concentration of the bacterial suspension was increased to three McFarland, the other strains were grown at one McFarland, and the degassing time was two minutes for all strains. Experiment 3: All strains were grown at one McFarland, and the tubes were degassed for three minutes. *E. bolteae* strain s28/42 was grown on two different media (YPG and BACT/ALERT^®^ FN Plus flask (bioMérieux, Lyon, France)). The BACT/ALERT FN Plus flasks for blood culture contain polymeric beads that adsorb antibiotics with peptones/biological extracts, anticoagulants, vitamins, amino acids, carbon sources, trace elements, and other complex amino acid and carbohydrate substrates in purified water, in a vacuum atmosphere of N_2_ and CO_2_.

Quantification of ethanol produced in each culture was carried out by headspace gas chromatography–mass spectrometry (HS-GC–MS) using a Turbomatrix HS110 sampler connected to a Clarus 690 chromatograph and a SQ8 T single quadrupole mass spectrometer (Perkin Elmer, Waltham, MA, USA). A standard ethanol curve was prepared with concentrations ranging from 0.5 mM to 100 mM spiked with isopropanol as an internal standard (5 mM). Samples were dispensed into headspace (HS) vials using 1 mL aliquot of each culture, then spiked with isopropanol. HS vials were heated at 60° C (for 10 min) and pressurized to 25 psi (1 min). The volatilized content was transferred to the GC (0.03 min, split 10/1). Alcohols were then separated through an Elite BAC2 column (30 m, 0.32 ID, Perkin Elmer) maintained at 70 degrees using helium as carrier gas (19.5 psi). A selected ion recording method was used to measure ethanol (31 *m/z*) and isopropanol (45 *m/z*). The data obtained were analyzed using Turbomass 6.1 software (Perkin Elmer).

### 2.6. Bioinformatic Analysis

The raw sequencing data for all samples were deposited into the NCBI Sequence Read Archive database (accession number: PRJEB62828). Noisy sequencing data were excluded, and chimeric sequences were identified and removed by Chimera Slayer. The clean data were clustered into operational taxonomic units (OTUs) at the 97% similarity threshold using UCLUST algorithm after the removal of singletons. The alpha, beta diversity, and linear discriminant analysis (LDA) were calculated using the Microbiome Analyst Platform (https://www.microbiomeanalyst.ca/; accessed on 17 March 2023) [55]. Shared taxa present in all groups were defined as the core microbiota.

### 2.7. Statistical Analysis

The results are expressed as the mean ± standard deviation. The normal distribution was performed using the Kolmogorov–Smirnov test. Two-tailed unpaired Student’s *t*-tests or Mann–Whitney tests were used when the data were normally or not normally distributed, respectively. Moreover, the Chi-squared test was used to compare the proportions of a sufficiently large number of species (˃20 in each contingency table cell). In contrast, Fisher’s exact and bilateral Barnard’s exact tests were used if the number of species was small (typically < 20 in one or more cells of the table). For gut microbiota analysis, differences in the relative abundances of OTUs of dominant bacteria were analyzed using Mann–Whitney U-tests. Statistical significance was accepted at *p* < 0.05. All analyses were performed with GraphPad Prism Software for Windows (GraphPad Software, San Diego, CA, USA) (version 9.0).

## 3. Results

### 3.1. Altered Diversity in Chronic HBV Samples by Culturomics

Of the 18 samples, 29,120 colonies were analyzed by MALDI–TOF to identify a total of 340 species belonging to 169 genera, and 6 phyla (chronic HBV samples (n = 8) yielded 240 species and 14,340 colonies (1793 ± 225 colonies per sample); conversely. control samples (n = 10) yielded 254 species, and 14,780 colonies (1478 ± 265 colonies per sample) were isolated (unpaired *t*-test, *p* = 0.0168). In addition, 154 species were shared between chronic HBV and control groups, while 86 species were uniquely detected in the chronic HBV group. A detailed list of the isolated bacterial species is mentioned in Supplementary File S1.

In chronic HBV samples, 5 bacterial phyla were isolated, with mostly *Bacillota* (148 species), followed by 34 *Bacteroidota*, 32 *Actinobacteria*, 16 *Proteobacteria*, and 2 *Synergistetes*. In control samples, 6 phyla were isolated, with a majority of *Bacillota* (143 species), followed by 50 *Bacteroidota*, 40 *Actinobacteria*, 12 *Proteobacteria*, 1 *Synergistetes*, and 1 *Verrucomicrobia* (Table S1).

**Figure 1:**
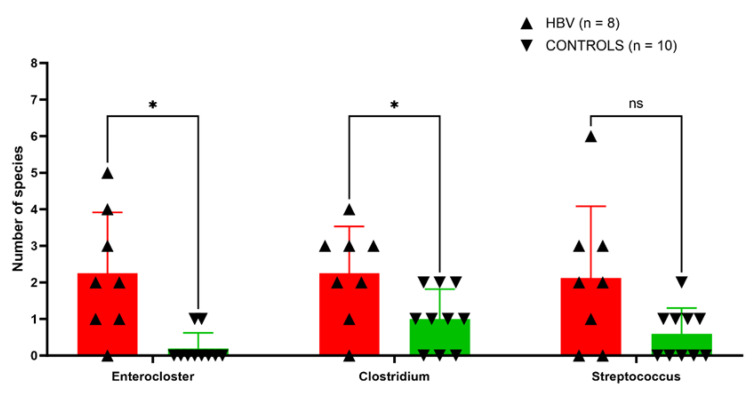
Boxplot showing the number of species per sample identified by culturomics in the three genera with a significant frequency difference between patients with chronic hepatitis B virus (HBV) infection (Red) versus the control group (Green). Two-tailed Mann–Whitney test; * *p* value < 0.05. ns: not statistically significant.

The 240 species isolated from chronic HBV samples belonged to 43 different families and 128 different genera. Among them, nine were the best-represented genera in terms of the diversity in the species, including *Bacteroides* (eleven species), *Enterococcus* (eleven), *Alistipes* (nine), *Streptococcus* (nine), *Clostridium* (eight), *Peptinophilus* (eight), *Enterocloster* (five), and *Prevotella* (five). In control samples, a total of 111 genera were identified, with the 7 most-represented genera including *Bacteroides* (15 species), *Enterococcus* (11), *Alistipes* (12), *Peptinophilus* (9), *Bifidobacterium* (7), *Limosilactobacillus* (7), and *Clostridium* (6) (Table S1). Thirty-four genera were only represented in the chronic HBV group and not in the control group. Three bacterial genera from the *Bacillota* and *Bacilli* phyla were found to have increased diversity (number of species of this genus per sample) in patients with chronic HBV infection, although only two genera, *Enterocloster* (*p* = 0.0016) and *Clostridium* (*p* = 0.041), showed significantly increased diversity (Table S1; Figure 1).

**Figure 2:**
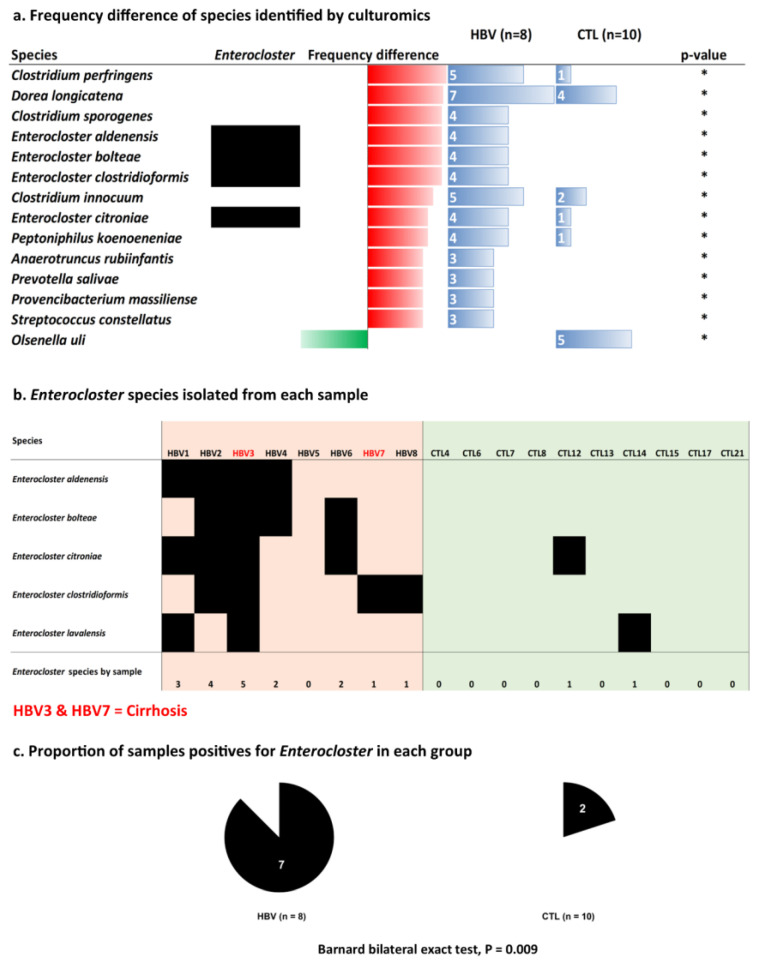
(**a**) Species with a significant frequency difference between chronic hepatitis B virus (HBV) and control (CTL) samples by the culturomics approach. (**b**) Detailed *Enterocloster* species isolated per sample. (**c**) Proportion of samples positive for *Enterocloster* species. Barnard’s bilateral exact test was used to test the *p*-value. * *p*-value < 0.01.

At the species level, the species with the highest significant difference in frequency belonging to class *Clostridia* were four *Enterocloster* (*E. bolteae*, *E. aldenensis*, *E. clostridioformis*, and *E. citroniae*) and three *Clostridium* (*C. perfringens*, *C. sporogenes* and *C. innocuum*) in the chronic HBV group. However, *Olsenella uli*, which belongs to Class *Coriobacteriia*, was more frequent in the control group (Figure 2a). The proportion of positive samples for *Enterocloster* was higher in the chronic HBV group (7/8 vs. 2/10, *p* = 0.009, bilateral Barnard’s exact test). Two patients with chronic HBV infection (HBV3, HBV7) were diagnosed with cirrhosis. HBV3 showed a greater diversity of *Enterocloster* species than the other samples (Figure 2b).

Overall, the hitherto unknown diversity was assessed and defined as the number of new species added to the species not previously known from the human gut by sample for culturomics analysis and as the number of unidentified OTU for metagenomics analysis [22]. The difference was not significant by culturomics for new species (0.75 ± 1.09 in chronic HBV vs. 1 ± 1.6 in controls, *p* = 0.96), as well as for previously known species which had not been previously found in humans (3.5 ± 1.41 in HBV vs. 2.36 ± 1.07 for controls, *p* = 0.0763) (Table S2).

By comparing the species isolated in the chronic HBV group to a recently published cultured human bacteria repertoire describing 3242 bacterial species, 81 shared species, and 5 species were uniquely defined in the HBV group. Moreover, 14 species were found in common between chronic HBV and control groups (Supplementary File S1). In addition, among the 240 species isolated in the chronic HBV group, 175 species were known in the human gut, 48 species were known in humans but not in the gut, 17 species were known but not previously found in humans, and 6 species were identified as new species (Table S3). In the control group, 11 unknown species were isolated from the human gut, among which 4 new genera were identified (Table S3), 13 were known but not previously found in humans, and 44 were already known in humans but not previously found in the gut.

### 3.2. Diversity Assessed by Metagenomics

The sequencing run expressed good quality monitoring parameters, as denoted in a cluster density of 965 K/MM2, passing filters of 53.6%, Q score of 2.3G 83.6%, and PhiX of 15%. Sequencing yielded 1,704,021 good-quality total reads (associated and non-associated reads) for the 18 samples included in our study (1,016,049 for chronic HBV and 687,972 for controls). The number of reads per sample was significantly higher in the chronic HBV group (127,006 ± 58,171) than in controls (68,797 ± 33,188) (*p* = 0.016).

**Figure 3:**
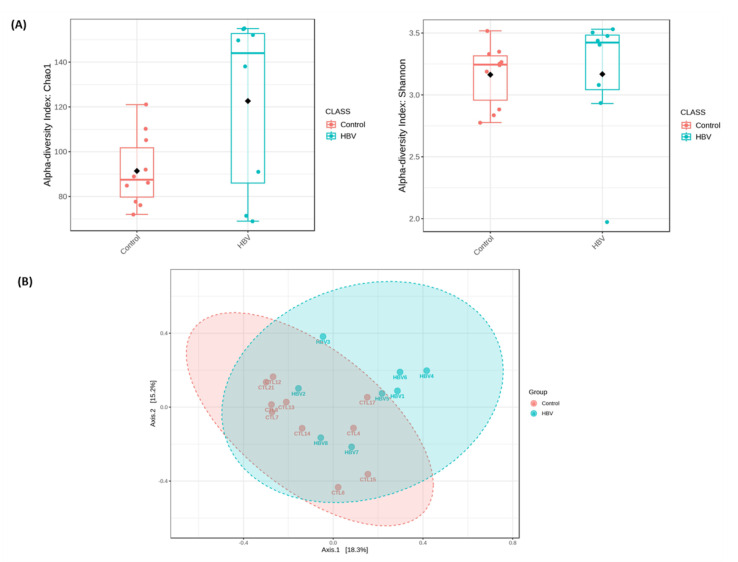
Comparison of alpha diversity and beta diversity in patients with chronic hepatitis B virus (HBV) and control groups. (**A**) Alpha diversity in box plots. Each boxplot represents the data range (whiskers), upper and lower quartiles (edges), the median (horizontal line), and the mean (black diamond). (**B**) Principal coordinate analysis (PCoA) plot of beta diversity.

**Figure 4:**
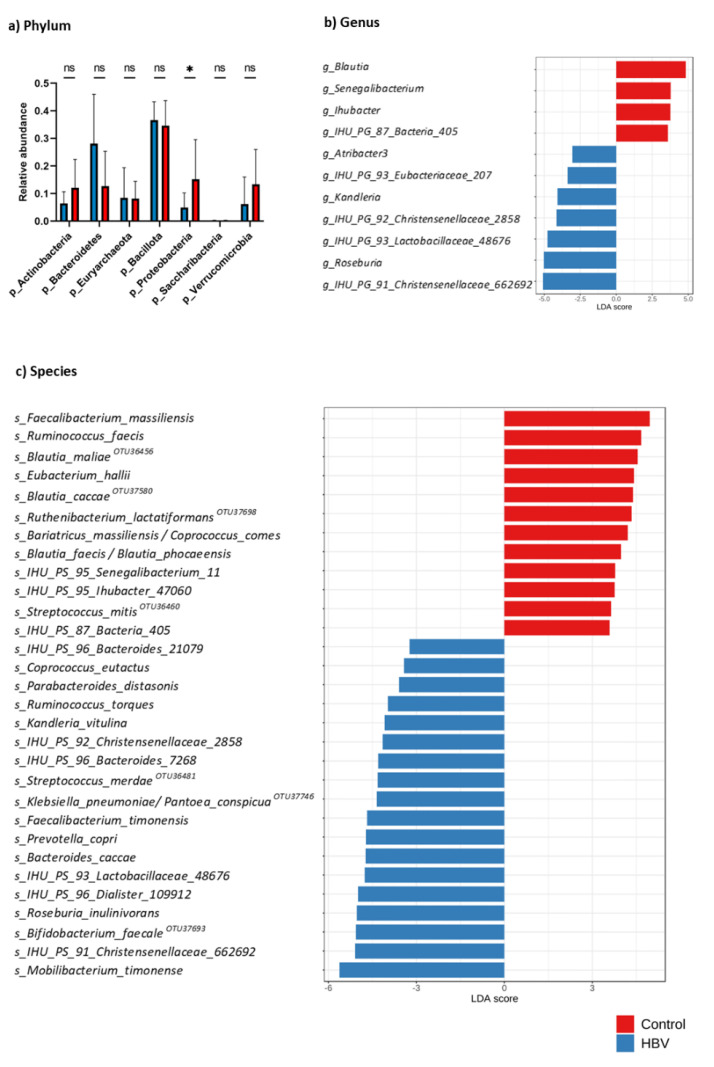
Relative abundance of gut microbiota in patients with chronic HBV infection (n = 8) and the control group (n = 10). (**a**) Barplot expressing abundant phyla. (**b**) Genus-level and (**c**) species-level were selected via LEfSe (LDA score > 2). Some OTUs correspond to several species (see Table S5). For clarity, only one clinically relevant species is represented on the graphic. HBV: Hepatitis B virus; LEfSe: linear discriminant analysis effect size; LDA: linear discriminant analysis; OTUs: operational taxonomic units. ns: not statistically significant. * *p* < 0.05.

The metagenomics analysis showed that in chronic HBV samples, 826,517 associated reads were dispersed throughout 7 phyla (*Actinobacteria*, *Bacteroidetes*, *Euryarchaeota*, *Bacillota*, *Proteobacteria*, *Candidatus Saccharibacteria*, and *Verrucomicrobia*). Control samples generated 507,865 associated reads and were divided into the same 7 phyla detected in the chronic HBV group. These reads matched 682 species in the HBV group and 715 in the control group. The richness and diversity of both groups were compared through the alpha diversity metrics shown in Figure 3. There was no significant difference in the Chao1 index (*p* = 0.168) or Shannon index (*p* = 0.315). Beta diversity showed significant differences in bacterial communities through principal coordinate analysis (PCoA) (R^2^ = 0.108, *p* = 0.009) (Figure 3). No significant difference existed between the groups, except for *Proteobacteria*, which showed a significant decrease in patients with chronic HBV infection, *p* = 0.034 (Figure 4). The chronic HBV group showed 76 genera and the control group showed 74 genera (*g_IHU_PG_93_Eubacteriaceae_207* and g_*Atribacter3* were absent from the control group) (Table S4). Highly significant different genera in both groups with LDA score ˃ 2.0 and *p* < 0.05 are illustrated in Figure 4. Interestingly, no significant difference was noticed regarding abundance for the same frequent genera detected by culturomics as *Clostridium* (*p* = 0.145) and *Streptococcus* (*p* = 0.260). *Enterocloster* was not recognized as an abundant taxon by LDA. Highly abundant OTUs assigned to the species level with LDA score ˃ 2.0 and *p* < 0.05 in both groups were shown in Figure 4.

**Figure 5:**
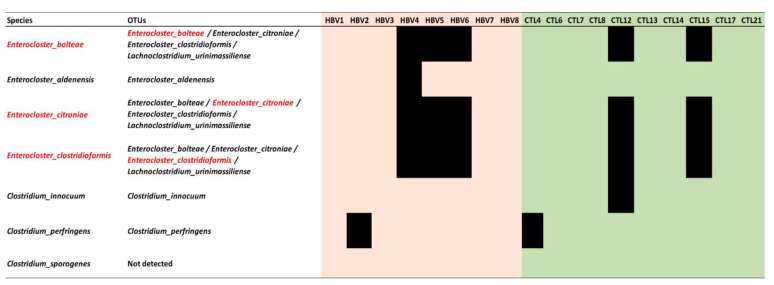
*Enterocloster* and *Clostridium* species detected in 16s RNA sequencing data file per sample. OTUs: Operational Taxonomic Units; HBV: hepatitis B virus infection; CTL: control. *E. bolteae*, *E. citroniae*, and *E. clostridioformis* (red words) were identified equivocally.

Notably, six out of all seven species belonging to *Enterocloster* and *Clostridium* genera were identified as multi-assigned OTUs in the dataset of 16S ribosomal RNA sequencing results (*Enterocloster_aldenensis*, *Enterocloster_bolteae*, *Enterocloster_citroniae*, *Enterocloster_clostridioformis*). *Enterocloster_bolteae* could not be identified as a single OTU. *Clostridium_innocuum* was identified as a single OTU, while *Clostridium_perfringens* was identified in single and multi-assigned OTUs. *C. sporogenes* was not identified at all in the samples. *Clostridium_innocuum* and *Clostridium_perfringens* species were unequivocally identified (only one species is known for this OTU). Three species were identified equivocally (Figure 5). Overall, none of the seven species with significant differences identified by culturomics had a considerable difference by sequencing. Similarly, no significant differences were noticed regarding the relative abundance of all OTUs attributed to *Enterocloster* or *Clostridium* at the genus level.

The metagenomics analysis showed a decreased aerotolerant alpha diversity (1.11 ± 0.40 in chronic HBV vs. 1.21 ± 0.49 in controls; *p* = 0.514) in addition to a decreased anerointolerant diversity in the chronic HBV group (2.85 ± 0.53 in HBV vs. 3.08 ± 0.31 in controls; *p* = 0.359). These results confirmed the specific decrease in anaerobic diversity found by culturomics, indicating loss of anaerobic species in patients with chronic HBV infection. The hitherto unknown diversity (unidentified OTUs) was consistently and significantly increased in the chronic HBV group (*p =* 0.0434). Additionally, at the prokaryotic level, 18.65% of all reads in the chronic HBV group were not assigned vs. 25.67% in control (*p* < 0.0001).

### 3.3. Missing Repertoire in Patients with Chronic HBV Infection

For identification of potential probiotic species, all of the bacterial species that were identified both by culturomics and metagenomics were considered in the control samples but not in HBV samples (Supplementary File S1). The common species between both approaches in the control group were six, including *Alistipes merdae*, *Christensenella massiliensis*, *Dialister succinatiphilus*, *Fenollaria timonensis*, *Mediterranea massiliensis*, and *Metaprevotella massiliensis.* However, the HBV group showed nine shared species between both approaches, including *Clostridium marseillense*, *Mogibacterium neglectum*, *Mogibacterium vescum*, *Pantoea agglomerans*, *Prevotella caccae*, *Prevotella copri*, *Terrisporobacter glycolicus*, *Weissella cibaria*, and *Weissella confusa.*

Comparing the species identified by metagenomics and culturomics in chronic HBV and control groups, 213 species were found exclusively in the control samples (Supplementary File S1). These species belonged overwhelmingly to the *Bacillota* phylum (89; 41.75%) and *Proteobacteria* (73; 34.27%), followed by a low number of species from the *Actinobacteria* (22; 10.33%), the *Bacteroidetes* (26; 12.21%), and *Euryarchaeota* (1; 0.47%) phyla. Among the missing repertoire, strikingly, 129 species (60.56%) were strictly anaerobic.

The literature was searched for each species to find a possible probiotic use for humans. Three species, *Limosilactobacillus oris*, *Propionibacterium freudenreichii*, and *Streptococcus oralis*, were found to have possible probiotic features (Table S6). Two of them (*L. oris* Q6189 *and S. oralis* Q4071) were isolated by culturomics in the control samples and are readily available in our laboratory’s Collection de Souches de l’Unité des Rickettsies (CSUR) collection.

### 3.4. Ethanol Quantification Produced by Enterocloster Species

**Figure 6:**
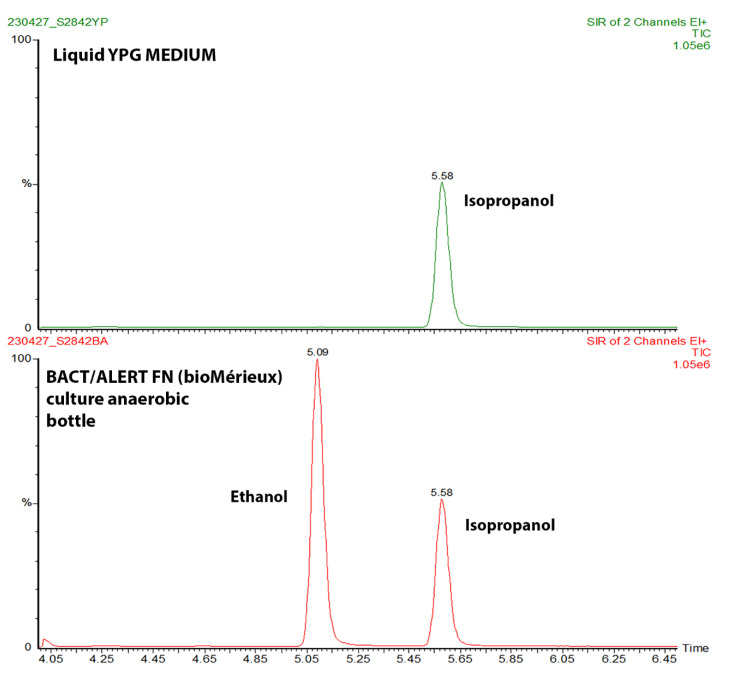
Detection of ethanol by gas chromatography–mass spectrometry (GC–MS) produced by isolated *Enterocloster* strain S28/42 in HBV using YPG or BACT/ALERT^®^ FN Plus culture media. The first chromatogram shows the analysis of *E. bolteae* strain s28/42 grown on YPG medium (10% glucose), in which bacterial growth was deficient and no ethanol was detected. The second chromatogram corresponds to the same strain grown on BACT/ALERT^®^ FN Plus, in which bacterial growth was evident within 24 h, and the presence of ethanol was observed at the peak at 5.09. The peak at 5.58 in both graphs corresponds to the isopropanol used as an internal standard.

The ethanol production of strains isolated from patients with chronic HBV infection was investigated (Table S7). Following two attempts in YPG and BACT/ALERT FN media, the third effort using a closed BACT/ALERT FN bottle to establish controlled anaerobiosis with an optimized medium (a technique developed during microbial culturomics) [48], showed significant ethanol production (Figure 6). No ethanol detection was observed for any of the *E. bolteae* strains or *E. citroniae* in experiment 1 and 2 (Table S7). However, in experiment 3, *E. aldenensis* strain s16/38 and *E. bolteae* strain s28/42 exhibited a weak ethanol production below 0.25 mM on YPG medium. Interestingly, *E. bolteae* strain s28/42 showed significantly higher ethanol production when growing on FN medium (Table S7).

## 4. Discussion

In this study, we report four enriched *Enterocloster* species, for the first time, in patients with chronic HBV infection using the culturomics approach rather than metagenomics. To our knowledge, no *Enterocloster* species were linked to chronic HBV infection.

The “culturomics” approach, whose effectiveness in analyzing gut microbiota has not yet been established [18], offers a significant benefit over metagenomics to exclude the enormous number of ingested bacteria that are killed in the upper gut by the acidic environment and bile salts [56] and to provide live strains on which further analysis can be performed. The popular method for examining the diversity of gut microbiota is metagenomics; however, the results of those studies has shown very low reproducibility, likely due to variations and biases in sampling, DNA extraction methodology, sequencing method, and data analysis technique [57]. The discordance between culturomics and metagenomics results has been a surprising finding since the very first development of this approach in 2009, with less than 20% common species [48]. Interestingly, the metagenomic approach detected a lower number of bacterial species than culturomics in a study conducted on a patient with resistant tuberculosis [58]. This demonstrates the depth bias of metagenomics. Moreover, it is a central point for the culturomics approach developed in our lab ten years ago to consider that all bacteria could grow [48]. Indeed, culturomics aims to reproduce the natural micro-environment and successfully discover hundreds of new bacterial species. However, several species and strains remain uncultured to date. However, the sample’s preparation could significantly impact culturability, including the mode of sampling, time of exposure to oxygen, and mode of freezing. For instance, using cryoprotectants could have dramatically improved culturability [59]. In addition, it was reported that culturability depends on the abundance of the bacterial species in the sample [31].

Eventually, both approaches seem to have several biases that could explain the disparities and complementarity [18,19]. Nevertheless, despite the discrepancy between metagenomics and culturomics [60,61], culturomics allowed the extension of gut microbiota’s known diversity and functions [62], as we proved by the results of this study. Furthermore, culturomics is a validated technique with consistent species identification that avoids some previously mentioned disadvantages of the metagenomic approach, such as inferior taxonomic resolution [19].

We intended to analyze ten samples per group by culturomics because it is a time-consuming approach in which one sample takes approximately four weeks to be analyzed. In addition, this small study was designed to be an exploratory study typically conducted to gain insights, explore potential relationships, and generate hypotheses for further investigation. Furthermore, exploratory research depends on the topic and the field. However, exploratory studies often come with certain limitations, and one common concern is the small sample size [63]. In our exploratory study, it is not only about the time consumed for every culture condition. It is also about the in-depth and accurate identification of the isolated colonies; as mentioned in the results section, from the 18 samples, 29,120 colonies were analyzed by MALDI–TOF to identify a total of 340 species (HBV samples (n = 8) yielded 240 species and 14,340 colonies (1793 ± 225 colonies per sample) vs. control samples (n = 10) yielded 254 species, and 14,780 colonies (1478 ± 265 colonies per sample) were isolated; unpaired *t*-test, *p* = 0.0168) (Supplementary File S1). Since power refers to the likelihood that we will find a significant result (an effect) in a studied sample or population [64]. Accordingly, several colonies and species identified per sample in our study made our results robust. The significant difference with low statistical power corresponded to dramatic and clear-cut differences. Hence, further studies with increased power (from more samples) are encouraged to confirm our results and decipher less important differences.

A total of 240 and 254 living, viable, and cultivable bacterial species were identified in the chronic HBV and control groups, respectively. Fifty-five species have been recognized as core microbiome detected by both approaches. Among them, 213 species formed a missing repertoire in patients with chronic HBV infection since they were found by both techniques exclusively in the healthy control group but not in the chronic HBV group. The global diversity, by culturomics, was significantly decreased alongside the aerointolerant species in the HBV group compared to controls. Metagenomic results showed the same diversity pattern. The hitherto unknown diversity was significantly increased by metagenomics in the chronic HBV group. Nevertheless, some studies using the metagenomic approach showed different results. Joo et al. reported higher alpha diversity in the HBV group [65], while Zheng et al. reported its decrease [66]. This could be attributed to differences in sample sizes and study populations.

Gut microbiota at the phylum level showed a non-significant increase in *Proteobacteria* and a decrease in *Actinobacteria* and *Bacteroidetes* in the HBV group by culturomics. However, metagenomics showed a significant reduction in *Proteobacteria* and a non-significant increase in *Bacteroidetes*. Indeed, several metagenomic studies supported our culturomics findings, which showed a continuous increase in the abundance of *Proteobacteria* in chronic HBV infection [11,14]. Interestingly, in agreement with Lagier et al. [18], most undetected human gut bacterial species grew in strict anaerobic (44%) or microaerophilic (5%) conditions, and 33% of them belonged to the phylum *Proteobacteria*. In contrast, only 9% belonged to the phylum *Bacteroidetes.* Those findings also support our culturomics results and align with the fact that our developed culturomics culture conditions were applied to cultivate those missing bacteria. However, the discrepancy in *Proteobacteria* abundance results between culturomics and metagenomics approaches could be attributed to the small sample size or other previously mentioned biases in the metagenomic approach. According to further research, *Bacteroidetes* and *Actinobacteria* in the chronic HBV group either increased or decreased [11,14,15]. Interestingly, both approaches reported an increase in *Bacillota*, which was in agreement with the previous studies [14,66]. Therefore, further exploration of gut microbiota dysbiosis in chronic HBV infection is warranted.

Among the top highly represented genera by culturomics, *Enterocloster* and *Clostridium* were significantly increased in the chronic HBV group. However, metagenomics showed a significant abundance of different genera, including *Roseburia*, *Kandleria*, and *Atribacter*, in the HBV group. Our results are different from most previous studies, which reported a decrease in *Clostridium* [11,16,17] and *Roseburia* [15,16] genera in chronic HBV infection. This could be attributed to the different sample size and diagnostic approaches used in this study. *Kandleria*, a genus from the family of *Erysipelotrichidae* [67], and *Atribacter*, a genus of the candidate phylum *Atribacterota* [68], have not been reported before in patients with liver disease, especially with chronic HBV infection. Although non-significantly, the increased abundance of Streptococcus in the chronic HBV group agreed with many previous studies [14,15,16,69].

*Enterocloster* is a genus recently identified in 2019 [32] as a reclassification of *Clostridium* genus, thanks to improved genomics and taxonomy. An unbiased study recently identified this new genus as the main provider of inoviruses [69], potentially pathogenic prophage viruses [70,71]. This genus comprises six validated species [32], including *E. aldenensis*, *E. asparagiformis*, *E. bolteae*, *E. citroniae*, *E. clostridioformis*, and *E. lavalensis*. Five *Enterocloster* species were isolated from chronic HBV samples; only two were in controls. Four species (*E. aldenensis*, *E. bolteae*, *E. citroniae* and *E. clostridioformis*) showed a significantly increased frequency in patients with chronic HBV infection. Notably, both *E. clostridioformis*/*E. bolteae* and *E. asparagiformis/E. lavalensis* were closely related based on phylogenetic, phylogenomic, and phenotypic perspectives [32]. However, culturomics alone could separately identify those species that were not distinguishable by sequencing but well-identified by MALDI–TOF, with high identification score > 2. A total of 18 OTUs were identified as *Enterocloster* species, and 448 OTUs were assigned for *Clostridium* species. Among 18 OTUs of *Enterocloster* species, seven OTUs were multi-assigned for *E. asparagiformis* and *E. lavalensis. E. bolteae* has been identified in three multi-assigned OTUs.

Species of *Enterocloster* genus have been associated with different diseases and dysbiosis. *Enterocloster aldenensis* was reported in intra-abdominal infections [72]. Additionally, a high carbohydrate-fermenting *Enterocloster* species such as *E. clostridioformis* was documented to be associated with clinical bacteremia cases [73] and was highly abundant in Type II diabetes [74] and Crohn’s disease [75]. Interestingly, *E. bolteae* was reported in different neurological diseases such as autism [38,76], multiple sclerosis [77], and neuromyelitis optica spectrum disorders [78]. According to a prior study, *E. bolteae* could produce microbially conjugated bile acids that contribute to the severity of Crohn’s disease and irritable bowel syndrome (IBS) [79]. Moreover, *E. bolteae* was identified as a mediator in fatty acid (FA) acylation to isoBAs (bile acids) [80]. *E. bolteae* has been reported in patients with fatty liver disease [37]. However, *E. bolteae* is not reported in chronic HBV infection; additionally, the role of its produced FA–isoBAs in host physiology or hepatic diseases is under investigation. Surprisingly, among *Entercloster* species, *E. citroniae* was described as an enriched purine-degrading species and considered a promising therapeutic prebiotic to reduce serum uric acid levels in a clinical trial performed on renal failure patients [81]. Moreover, patients on peritoneal dialysis who experienced a restriction of advanced glycation end products diet (to decrease cardiovascular disease incidence) showed an increase in *E. citroniae*, suggesting its beneficial role [82]. As a result, the increased prevalence of the *Enterocloster* species mentioned in chronic HBV infection necessitates more research into their potential role in liver disease progression, particularly HBV-related complications.

Our culturomics results showed significant abundance in three *Clostridium* species, including *C. perfringens*, *C. innocuum*, and *C. sporogenes* in the HBV group. In fact, the genus *Clostridium* has recently been taxonomically clarified [83], making it possible to specify the associations between this genus and several diseases. In particular, *C. perfringens* is associated with necrotizing enteritis, enterotoxemia, and gas gangrene [84]. Moreover, cirrhotic patients infected with *C. perfringens* manifested poor prognosis [85]. *Clostridium innocuum* was described as an extraintestinal pathogen causing bacteremia, endocarditis, osteomyelitis, and peritonitis and may also cause a *C. difficile*-like antibiotic-associated diarrheal illness [86]. Additionally, *C. sporogenes* was able to transform tryptophan into indole-3-propionic acid affecting intestinal permeability and was found to be negatively correlated with several metabolic diseases [87]. Therefore, the potential role of those particular species deserves further investigation in patients with chronic HBV infection.

Additionally, our study reported different abundant species in the HBV group by metagenomics such as *Coprococcus_eutactus* (OTU731), *Parabacteroides_distasonis* (OTU38338), *Ruminococcus_torques* (OTU38812), *Kandleria_vitulina* (OTU34133), and multiassigned OTUs for *Streptocoocus* (OTU36481) and *Bifidobacterium* (OTU37693) species. Surprisingly, both culturomics and metagenomics approaches identified nine and six species in HBV and control samples, respectively. However, those species were not significantly frequent or abundant, except for *P. copri* (OTU38502), which was significantly abundant in the HBV group by metagenomics. In addition, previous studies had also reported an increase in the *Prevotella* genus in chronic HBV infection [12,15]. *Prevotella* was reported to be related explicitly to the immunotolerant phase of HBV infection [88], indicating that members of *Prevotella* genus could play a vital role in viral escape from the host immune system.

Briefly, the differences between our findings using culturomics and metagenomic approaches and previous HBV-associated gut dysbiosis studies have been summarized in Table S8. Interestingly, most previous studies reported gut dysbiosis to the genus level, and only a few reported the species level. The characteristics of the chronic HBV-associated dysbiosis in our study showed a depletion of aerointolerant diversity and enrichment in potentially pathogenic *Enterocloster* and *Clostridium* species. Unlike earlier research, our study found an increase in *Clostridium* species. However, most previous studies agreed on a significant abundance of *Streptococcus* and *Bacteriode.* Moreover, one study reported a significant abundance in the same three species reported in this study, including *Dorea longicatena*, *C. citroniae*, and *P. salivae* [89]. Interestingly, no study has reported *E. bolteae* abundance in chronic HBV infection. The discordant results could be related to various factors, including sample size, the study populations, different disease stages, and various diagnostic techniques (culturomics with different culture conditions), while previous studies utilized solely the metagenomic approach. In summary, no method is ideal, and culturomics and metagenomics are necessary and complementary for explaining the dysbiosis associated with chronic HBV infection.

Under anaerobic conditions, it has been reported that *Escherichia*, *Bacteroides*, *Bifidobacterium*, and *Clostridium* genera can produce ethanol by fermentation from consumed carbohydrates [90]. Moreover, *E. bolteae* species was particularly interesting because bacteria from the *Clostridia* class carried genes encoding for ethanol production pathways [37]. Interestingly, *E. bolteae* was described as an opportunistic pathogen in humans [90]. Additionally, it caused liver damage through endogenous ethanol synthesis in patients with non-alcoholic fatty liver disease (NAFLD) by increasing the permeability of the gut epithelial barrier [91]. Consequently, *E. bolteae* strains were re-cultured, but the inoculum concentration increased to three MacFarland (experiment 2) to promote growth. However, no ethanol was detected in any *E. bolteae* strain, but slight bacterial growth was observed due to the turbidity of the medium. We speculated that this might be a problem linked to the tube’s anaerobic environment; therefore, the degassing time was increased to three minutes. The results of experiment 3 showed ethanol production from all the strains. Surprisingly, *E. bolteae* strain s28/42, grown in BACT/ALERT^®^ FN, produced the most ethanol. The previous deficit in *E. bolteae* growth could be attributed to the volume of the liquid medium (40 mL in BACT/ALERT^®^ FN vs. 10 mL in YPG medium). The ethanol production in the FN medium was achieved due to its higher nutritional content than the YPG medium.

Notably, intestinal microecological modulators are now used in clinical practice to treat HBV-related liver failure, particularly in patients with hepatic encephalopathy [92,93]. However, the therapeutic efficacy varies considerably due to the differences in gut microbiota composition. FMT is a promising technique to restore healthy microbiota, improve the current treatment protocol, and prevent chronic HBV-associated complications [94], as in cirrhotic patients and hepatic encephalopathy [95]. Tidjani et al. suggested that the ideal situation would be to culture the symbionts absent from patients but present in healthy control to define and accurately replicate a known and efficient mixed fecal microbiota [22]. Interestingly, on comparing the species identified in our study by metagenomics and culturomics in both HBV and control groups, 213 species were found exclusively in the control samples (Supplementary File S1). These species constituted the missing repertoire in patients with chronic HBV infection and could be used for FMT. Nevertheless, due to the limited number of participants and a lack of randomized clinical trials, further well-designed trials are required to confirm the initial assumptions and promote clinical practicability in the future [96].

Furthermore, three species of possible probiotic features were identified in our study, including *L. oris*, *P. freudenreichii*, and *S. oralis*. Some *L. oris* strains were reported as bacteriocin-producing strains used as protective cultures [97]. A probiotic supplement of *P. freudenreichii* was found to reduce the biologically effective dose of aflatoxin exposure and decrease the risk of liver cancer in a previous clinical trial [98]. However, *S. oralis* was involved in oral health by creating a healthy balance of beneficial bacteria while helping reduce its undesirable counterpart [99]. Interestingly, the role of probiotics in chronic HBV-associated complications such as encephalopathy has been proven [27,28]. Since the usage of the missing repertory of probiotics necessitates the use of viable isolated strains, the ability of the culturomics approach was demonstrated to play a critical role in the cultivation of such gut microbiota.

In this context, the present results suggest endogenous alcohol production by gut microbiota might participate in chronic HBV infection, as recently described in non-alcoholic steato-hepatitis (NASH) patients [100]. Additionally, microbial culturomics allowed us to obtain live bacterial species that could contribute to the pathophysiology of the disease.

Nevertheless, it is essential to acknowledge the limitations of our study in terms of small sample size and sample pre-freezing. The low number of samples in our exploratory study is compensated by the huge number of colonies by sample, allowing us to have a deep and robust characterization of each sample. Unfortunately, we did not use cryoprotectant for sample pre-freezing in this study, which is a possible limitation. Cryoprotectants such as trehalose, glycerol, and/or skimmed milk are proven to improve the culturability of any frozen fecal sample [59]. However, it cannot be ruled out that cryoprotectants may alter the microbial profile of any sample. Moreover, among the transport medium’s constitutions used for the samples of this study, two antioxidants, namely glutathione and uric acid, were included to maintain the cultivability of fecal anaerobes [101]. Therefore, our results are still relevant since the anaerobic spore-forming *Clostridium* species were recovered and isolated successfully from the frozen samples.

Future research should consider more diverse samples and focus on elucidating the mechanisms by which *E. bolteae* might contribute to liver inflammation and HBV disease progression and on exploring interventions to restore healthy gut microbiota in chronic HBV infection.

Finally, our results open new insight into microbiota’s potential role in the pathophysiology of chronic HBV infection, paving the way for further research regarding microbiome-targeted therapeutic options such as probiotics and FMT.

## Data Availability

The metagenomic files are available online under bio project number PRJEB62828. All the cultured species in this study are preserved and available for further investigation. They are deposited in the Collection de Souches de l’Unité des Rickettsiesand (CSUR) collection numbers as *Enterocloster bolteae* Q5636, *Clostridium sporogenes* Q5441, and *Clostridium sporogenes* Q5652. Probiotic species are available and deposited in our CSUR collection: *Limosilactobacillus oris* Q6189, *Propionibacterium freudenreichii* Q7880, and *Streptococcus oralis* Q4071.

## Supplementary Materials

The following supporting information can be downloaded at: https://www.mdpi.com/article/10.3390/microorganisms11102437/s1. References [102,103,104,105,106,107,108,109,110,111,112,113,114,115,116,117,118,119,120,121,122,123,124,125,126,127,128,129,130] are cited in the supplementary materials. Table S1: Bacterial diversity by culturomics. Table S2: Comparison of the cultured gut bacterial global and hitherto diversity between chronic HBV patients and control. Table S3: Unique and putative new species isolated in chronic HBV patients and control groups. Table S4: Relative genus abundance of gut microbiota in chronic HBV patients and control groups. Table S5: OTUs corresponding to species level. Table S6: Potential probiotics identified by culturomics and metagenomics in the control group and their possible effects. Table S7: Concentrations of ethanol produced by different *Enterocloster* species in the three tested conditions using gas chromatography-mass spectrometry (GC-MS). Table S8: Microbiota changes in previous case-control studies conducted on HBV-related diseases. File S1: Culturomics results.
