# Supplementary material for: Ethanol-Producing Enterocloster bolteae Is Enriched in Chronic Hepatitis B-Associated Gut Dysbiosis: A Case–Control Culturomics Study
Source: Microorganisms. 2023 Sep 28;11(10):2437. doi: 10.3390/microorganisms11102437 (PMC10608849; doi:10.3390/microorganisms11102437)
Supplement: Supplementary file 1 [file microorganisms-11-02437-s001.zip › Table_S2.pdf]

Table S2. Comparison of the cultured gut bacterial global and hitherto diversity between chronic HBV patients and control.

**Global diversity per sample (Mean + SD)**	**HBV (n=8)**	**Controls (n=10)**	***P*-value**
Number of phyla	4.25±0.43	4.18±0.39	>0.9999 [a]
Number of genera	40.88±9.57	35.55±9.55	0.2721 [b]
Total number of species	60.63±13.21	51.55±13.53	0.1856 [b]
Number of HG species	48.25±12.71	41±11.68	0.24 [b]
Number of HNG species	8.75±5.12	8.18±3.04	0.7785 [b]
Number of NH species *	3.5±1.41	2.36±1.07	0.1058 [a]
Number of new species *	0.75±1.09	1±1.6	0.9581 [a]

SD: Standard deviation; **HG:** species: species previously isolated in the human gut; **HNG:** species previously isolated in humans but not in the gut; **NH:** species not previously isolated in humans; [a] Two-tailed Mann-Whitney test; [b] Two-tailed unpaired t-test. * The hitherto unknown diversity is represented by the number of new species and the number of NH species.
