# Supplementary material for: Ethanol-Producing Enterocloster bolteae Is Enriched in Chronic Hepatitis B-Associated Gut Dysbiosis: A Case–Control Culturomics Study
Source: Microorganisms. 2023 Sep 28;11(10):2437. doi: 10.3390/microorganisms11102437 (PMC10608849; doi:10.3390/microorganisms11102437)
Supplement: Supplementary file 1 [file microorganisms-11-02437-s001.zip › Table_S3.pdf]

Table S3. Unique and putative new species isolated in chronic HBV patients and control groups.

Unique Species	Phylum	Family	Genera	Sample of origin	Collection number	New Species or Known	Origin	Isolation technique	Site	Year
Clostridium caccae	*Bacillota*	*Clostridiaceae*	*Clostridium*	HBV	CSUR P3106	New species IHU	Human	Culturomics IHU*	Gut, duodenum	2016
Dorea provencensis	*Bacillota*	*Lachnospiraceae*	*Dorea*	HBV	CSUR P4042	New species IHU	Human	Culturomics IHU	Gut, stools	2017
Niallia alba	*Bacillota*	*Bacillaceae*	*Nialla*	HBV	CSUR P3072	New species [1]	Food	Culture	digestive syrup	2022
Oribacterium massiliense	*Bacillota*	*Lachnospiraceae*	*Oribacterium*	HBV	CSUR P4859	New species IHU	Human	Culturomics IHU	Gut, stools	2017
Transplantifaecale massiliensis	*Bacillota*	*Clostridiaceae*	*Transplantifaecale*	HBV	CSUR P8228	New genus IHU	Human	Culturomics IHU	Gut, stools	2018
Agathobaculum massiliense	*Bacillota*	*Oscillospiraceae*	*Agathobaculum*	HBV/Control	CSUR P4005	New species IHU [2]	Human	Culturomics IHU	Gut	2023
Alistipes merdae	*Bacteroidota*	*Rikenellaceae*	*Alistipes*	Control	CSUR P8752	New species IHU	Human	Culturomics IHU	Gut	2018
Cutibacterium eggermontii	*Actinobacteria*	*Propionibacteriaceae*	*Cutibacterium*	Control	CSUR P8801	New species	Human	Culture IGR**	Gut	2017
Guopingia tenuis	*Bacillota*	*Christensenellaceae*	*Guopingia*	Control	KCTC 25142	Known [3]	Human	Culture (hGMB)***	Gut	2021
Lachnospira massiliensis	*Bacillota*	*Lachnospiraceae*	*Lachnospira*	Control	CSUR Q7423	New species IHU	Human	Culturomics IHU	Gut, stools	2022
Ligilactobacillus massiliensis	*Bacillota*	*Lactobacillaceae*	*Ligilactobacillus*	Control	CSUR Q7487	New species IHU	Human	Culturomics IHU	Gut, stools	2022
Mbogningella massiliensis	-	-	*Mbogningella*	Control	CSUR P3669	New genus IHU	Human	Culturomics IHU	Gut, stools	2016
Neobeduinibacterium massiliensis	*Bacillota*	*Christensenellaceae*	*Neobeduinibacterium*	Control	CSUR P4262	New genus IHU	Human	Culturomics IHU	Gut, stools	2017
Olsenella merdae	*Actinobacteria*	*Atopobiaceae*	*Olsenella*	Control	CSUR P8424	New species IHU	Human	Culturomics IHU	Gut, stools	2018
Phamibacteria massiliensis	*Actinomycetota*	*Eggerthellaceae*	*Phamibacteria*	Control	CSUR Q7649	New genus IHU	Human	Culturomics IHU	Gut, stools	2022
Pusillibacter massiliensis	*Bacillota*	*Oscillospiraceae*	*Pusillibacter*	Control	CSUR Q7486	New species IHU	Human	Culturomics IHU	Gut, stools	2022
Ruminococcoides bili	*Bacillota*	*Oscillospiraceae*	*Ruminococcoides*	Control	DSM 110008	New genus [4]	Human	Culture	Gut, bile	2021

CSUR: Collection de Souches de l´Unité des Rickettsies; **KCTC:** Korean Collection for Type Cultures; **DSM:** Deutsche Sammlung von Mikroorganismen und Zellkulturen ; **HBV:** Hepatitis B virus infection**.*** **IHU:** l'Institut Hospitalo-Universitaire; ****IGR:** Institute Gustave Roussy; ***Human Gut Microbial Biobank (hGMB; homepage: hgmb.nmdc.cn)

[1] Thorat, V.; Kirdat, K.; Tiwarekar, B.; Dhanavade, P.; Karodi, P.; Shouche, Y.; Sathe, S.; Lodha, T.; Yadav, A. Paenibacillus Albicereus Sp. Nov. and Niallia Alba Sp. Nov., Isolated from Digestive Syrup. *Arch Microbiol* **2022**, *204*, 127, doi:10.1007/s00203-021-02749-x.

[2] Magdy Wasfy, R.; Zoaiter, M.; Bilen, M.; Tidjani Alou, M.; Lo, C.I.; Bellali, S.; Caputo, A.; Alibar, S.; Andrieu, C.; Raoult, D.; et al. Description of Agathobaculum Massiliense Sp. Nov., a New Bacterial Species Prevalent in the Human Gut and Predicted to Produce Indole and Tryptophan Based on Genomic Analysis. *Antonie Van Leeuwenhoek* **2023**, doi:10.1007/s10482-023-01824-1.

[3] Liu, C.; Du, M.-X.; Abuduaini, R.; Yu, H.-Y.; Li, D.-H.; Wang, Y.-J.; Zhou, N.; Jiang, M.-Z.; Niu, P.-X.; Han, S.-S.; et al. Enlightening the Taxonomy Darkness of Human Gut Microbiomes with a Cultured Biobank. *Microbiome* **2021**, *9*, 119, doi:10.1186/s40168-021-01064-3.

[4] Molinero, N.; Conti, E.; Sánchez, B.; Walker, A.W.; Margolles, A.; Duncan, S.H.; Delgado, S. Ruminococcoides Bili Gen. Nov., Sp. Nov., a Bile-Resistant Bacterium from Human Bile with Autolytic Behavior. *Int J Syst Evol Microbiol* **2021**, *71*, doi:10.1099/ijsem.0.004960.
