# Supplementary material for: Ethanol-Producing Enterocloster bolteae Is Enriched in Chronic Hepatitis B-Associated Gut Dysbiosis: A Case–Control Culturomics Study
Source: Microorganisms. 2023 Sep 28;11(10):2437. doi: 10.3390/microorganisms11102437 (PMC10608849; doi:10.3390/microorganisms11102437)
Supplement: Supplementary file 1 [file microorganisms-11-02437-s001.zip › Table_S6.pdf]

Table S6. Potential probiotics identified by culturomics and metagenomics in the control group and their possible effects.

Species	Strain	Phylum	Status	Effect	Reference
Limosilactobacillus oris	-	*Bacillota*	Clinical trial	Urogenital health	[1]
Limosilactobacillus oris	P49	*Bacillota*	Research	Antibacterial activity	[2]
Propionibacterium freudenreichii	ET-3	*Actinobacteria*	Clinical trial	Inflammatory bowel disease (IBD)	[3]
Propionibacterium freudenreichii subsp. shermanii	-	*Actinobacteria*	Clinical trial	Liver cancer	[4]
Propionibacterium freudenreichii subsp. shermanii	-	*Actinobacteria*	Research	Anti-E. coli	[5]
Propionibacterium freudenreichii subsp. shermanii	CDB 10015	*Actinobacteria*	Research	Produces Vitamin B12	[6]
Propionibacterium freudenreichii subsp. shermanii	HA-182	*Actinobacteria*	Commercial	Supports digestion and immunity	[7]
Propionibacterium freudenreichii subsp. shermanii	JS	*Actinobacteria*	Clinical trial	Enhancement of Vaccine Responses	[8]
Propionibacterium freudenreichii subsp. shermanii	JS	*Actinobacteria*	Clinical trial	Alleviating IBD symptoms	[9]
Propionibacterium freudenreichii subsp. shermanii	JS	*Actinobacteria*	Clinical trial	Anti-H. pylori	[10]
Propionibacterium freudenreichii subsp. shermanii	JS	*Actinobacteria*	Clinical trial	Anti-inflammatory activity	[11]
Propionibacterium freudenreichii subsp. shermanii	JS (DSM 7076)	*Actinobacteria*	Clinical trial	Prevention of Allergic Diseases	[12]
Streptococcus oralis	KJ3	*Bacillota*	Commercial	Oral health	[13]
Streptococcus oralis	KJ3	*Bacillota*	Research	Oral health	[14]

1. Borges, S.; Silva, J.; Teixeira, P. The Role of Lactobacilli and Probiotics in Maintaining Vaginal Health. *Arch Gynecol Obstet* **2014**, *289*, 479–489, doi:10.1007/s00404-013-3064-9.
2. Grosu-Tudor, S.-S.; Stancu, M.-M.; Pelinescu, D.; Zamfir, M. Characterization of Some Bacteriocins Produced by Lactic Acid Bacteria Isolated from Fermented Foods. *World J Microbiol Biotechnol* **2014**, *30*, 2459–2469, doi:10.1007/s11274-014-1671-7.
3. Uchida, M.; Mogami, O.; Matsueda, K. Characteristic of Milk Whey Culture with Propionibacterium Freudenreichii ET-3 and Its Application to the Inflammatory Bowel Disease Therapy. *Inflammopharmacology* **2007**, *15*, 105–108, doi:10.1007/s10787-007-1557-5.
4. El-Nezami, H.S.; Polychronaki, N.N.; Ma, J.; Zhu, H.; Ling, W.; Salminen, E.K.; Juvonen, R.O.; Salminen, S.J.; Poussa, T.; Mykkänen, H.M. Probiotic Supplementation Reduces a Biomarker for Increased Risk of Liver Cancer in Young Men from Southern China. *Am J Clin Nutr* **2006**, *83*, 1199–1203, doi:10.1093/ajcn/83.5.1199.
5. Campaniello, D.; Bevilacqua, A.; Sinigaglia, M.; Altieri, C. Screening of Propionibacterium Spp. for Potential Probiotic Properties. *Anaerobe* **2015**, *34*, 169–173, doi:10.1016/j.anaerobe.2015.06.003.
6. Quesada-Chanto, A.; Schmid-Meyer, A.C.; Schroeder, A.G.; Carvalho-Jonas, M.F.; Blanco, I.; Jonas, R. Effect of Oxygen Supply on Biomass, Organic Acids and Vitamin B12 Production by Propionibacterium Shermanii. *World Journal of Microbiology and Biotechnology* **1998**, *14*, 843–846, doi:10.1023/A:1008868907251.
7. Chan, P.L.; Lauw, S.; Ma, K.L.; Kei, N.; Ma, K.L.; Wong, Y.O.; Lam, H.Y.; Ting, Y.Y.; Yau, T.K.; Nong, W.; et al. ProBioQuest: A Database and Semantic Analysis Engine for Literature, Clinical Trials and Patents Related to Probiotics. *Database* **2022**, *2022*, baac059, doi:10.1093/database/baac059.
8. Reents, R.; Dekkers, J.C.; Schaeffer, L.R. Genetic Evaluation for Somatic Cell Score with a Test Day Model for Multiple Lactations. *J Dairy Sci* **1995**, *78*, 2858–2870, doi:10.3168/jds.S0022-0302(95)76916-8.
9. Kajander, K.; Hatakka, K.; Poussa, T.; Färkkilä, M.; Korpela, R. A Probiotic Mixture Alleviates Symptoms in Irritable Bowel Syndrome Patients: A Controlled 6-Month Intervention. *Aliment Pharmacol Ther* **2005**, *22*, 387–394, doi:10.1111/j.1365-2036.2005.02579.x.
10. Myllyluoma, E.; Veijola, L.; Ahlroos, T.; Tynkkynen, S.; Kankuri, E.; Vapaatalo, H.; Rautelin, H.; Korpela, R. Probiotic Supplementation Improves Tolerance to Helicobacter Pylori Eradication Therapy--a Placebo-Controlled, Double-Blind Randomized Pilot Study. *Aliment Pharmacol Ther* **2005**, *21*, 1263–1272, doi:10.1111/j.1365-2036.2005.02448.x.

11. Hatakka, K.; Holma, R.; El-Nezami, H.; Suomalainen, T.; Kuisma, M.; Saxelin, M.; Poussa, T.; Mykkänen, H.; Korpela, R. The Influence of Lactobacillus Rhamnosus LC705 Together with Propionibacterium Freudenreichii Ssp. Shermanii JS on Potentially Carcinogenic Bacterial Activity in Human Colon. *Int J Food Microbiol* **2008**, *128*, 406–410, doi:10.1016/j.ijfoodmicro.2008.09.010.
12. Kukkonen, K.; Savilahti, E.; Haahtela, T.; Juntunen-Backman, K.; Korpela, R.; Poussa, T.; Tuure, T.; Kuitunen, M. Probiotics and Prebiotic Galacto-Oligosaccharides in the Prevention of Allergic Diseases: A Randomized, Double-Blind, Placebo-Controlled Trial. *J Allergy Clin Immunol* **2007**, *119*, 192–198, doi:10.1016/j.jaci.2006.09.009.
13. Chan, P.L.; Lauw, S.; Ma, K.L.; Kei, N.; Ma, K.L.; Wong, Y.O.; Lam, H.Y.; Ting, Y.Y.; Yau, T.K.; Nong, W.; et al. ProBioQuest: A Database and Semantic Analysis Engine for Literature, Clinical Trials and Patents Related to Probiotics. *Database* **2022**, *2022*, baac059, doi:10.1093/database/baac059.
14. Hillman, J.D.; McDonell, E.; Hillman, C.H.; Zahradnik, R.T.; Soni, M.G. Safety Assessment of ProBiora3, a Probiotic Mouthwash: Subchronic Toxicity Study in Rats. *Int J Toxicol* **2009**, *28*, 357–367, doi:10.1177/1091581809340705
