# Supplementary material for: Ethanol-Producing Enterocloster bolteae Is Enriched in Chronic Hepatitis B-Associated Gut Dysbiosis: A Case–Control Culturomics Study
Source: Microorganisms. 2023 Sep 28;11(10):2437. doi: 10.3390/microorganisms11102437 (PMC10608849; doi:10.3390/microorganisms11102437)
Supplement: Supplementary file 1 [file microorganisms-11-02437-s001.zip › Table_S7.pdf]

Table S7. Concentrations of ethanol produced by different *Enterocloster* species in the three tested conditions using gas chromatography-mass spectrometry (GC-MS).

Experiment	Strain	Sample	Species	EtOH (mM)	EtOH mL/L	EtOH g/L	EtOH %(vol)
McFarland 1; time 2 min	s28 42	HBV6	*Enterocloster bolteae*	0	0	0	0
McFarland 1-3; time 2 min	S16 60	HBV1	*Enterocloster citroniae*	0	0	0	0
	S17 44	HBV2	*Enterocloster citroniae*	0	0	0	0
	S28 35	HBV6	*Enterocloster citroniae*	0	0	0	0
	s28 42	HBV6	*Enterocloster bolteae*	0	0	0	0
McFarland 1; time 3 min	s16 38	HBV1	*Enterocloster aldensis*	<0.25	0	0	0
	s20 32	HBV4	*Enterocloster bolteae*	27	1.5764	1.2	0.2
	s28 42	HBV6	*Enterocloster bolteae**	200	11.6603	9.2	1.2
	s28 42	HBV6	*Enterocloster bolteae*	<0.25	0	0	0

*This stain has been grown on FN commercial medium supplemented with BACT/ALERT culture bottle unlike all other strains which were grown on YPG medium.
