# Supplementary material for: Ethanol-Producing Enterocloster bolteae Is Enriched in Chronic Hepatitis B-Associated Gut Dysbiosis: A Case–Control Culturomics Study
Source: Microorganisms. 2023 Sep 28;11(10):2437. doi: 10.3390/microorganisms11102437 (PMC10608849; doi:10.3390/microorganisms11102437)
Supplement: Supplementary file 1 [file microorganisms-11-02437-s001.zip › Table_S8.pdf]

Table S8 Microbiota changes in previous case-control studies conducted on HBV-related diseases.

Author	Country	Cohort (n)	Methodology	Microbiota changes
**Lu et al., 2011** [1]	China	HBV carriers (n=30)<br>CHB (n=31)<br>CHB-LC (n = 31)<br>Healthy Control (n= 32)	qPCR	**Phyla:**<br>*Bacteroidetes* ↓<br>*Firmicutes* ↓<br>**Genera:**<br>*Bifidobacterium* ↓<br>*Lactobacillus* ↓<br>*Pediococcus* ↓<br>*Leuconostoc* ↓<br>*Weissella* ↓<br>*Clostridium* ↓
**Deng et al., 2019** [2]	China	Total CHB-LC (n=80)<br>Healthy Control (n=20)	16S rRNA sequencing	**Phyla**<br>*Firmicutes/Bacteroidetes* ↑<br>**Genera:**<br>*Veillonella* ↑<br>*Megamonas* ↓
**Yun et al., 2019** [3]	South Korea	HBV carriers (n=36)<br>Normal ALT (n=26)<br>High ALT (n=8)<br>Healthy control (n=76)	16S rRNA sequencing	**Genera:**<br>*Anaerostipes* ↑<br>*Megasphaera* ↑<br>*Desulfovibrio* ↑<br>*Acidaminococcus* ↓
**Xu et al., 2012** [4]	China	CHB (n =16),<br>CHB-LC (n = 16),<br>Healthy Control (n = 15)	PCR-DGGE and qPCR	**Species:**<br>*B. dentinum,* ↑<br>*B.catenulatum* ↓<br>*B. longum* ↓

**Wang et al., 2020** [5]	China	CHB with fibrosis (n=69)<br>Healthy Control (n = 21)	16S rRNA sequencing	***Genera:***<br>*Prevotella* ↑<br>*Bacteroides* ↓<br>*Ruminococcus* ↓
**Wang et al., 2017** [6]	China	CHB (n = 85):<br>Healthy Control (n = 22)	16S rRNA sequencing	**Genera:**<br>*Alistipes* ↓<br>*Actinomyces* ↑<br>*Asaccharobacter* ↓<br>*Bacteroides* ↓<br>*Butyricimonas* ↓<br>*Escherichia/Shigella* ↓<br>*Parabacteroides* ↓<br>*Ruminococcus* ↓<br>*Megamonas* ↑
**Wang et al., 2021** [7]	China	CHB(n=252)<br>CHB-LC (n = 162)<br>HBV-ACLF (n=212)<br>Healthy controls (n=877)	16S rRNA and metagenomic	**Genera:**<br>*Enterococcus* ↑<br>*Faecalibacterium* ↑<br>**Species:**<br>*Enterococcus faecium* ↑<br>*Lactobacillus casei/paracasei* ↑<br>*Ruminococcus obeum* ↑<br>*Dorea longicatena* ↑<br>*Clostridium citroniae* ↑<br>*Faecalibacterium prausnitzii* ↑<br>*Parabacteroides merdae*↑<br>*Alistipes senegalensis* ↑<br>*Streptococcus vestibularis* ↑<br>*Prevotella salivae* ↑<br>*Prevotella histicola* ↑

				Streptococcus parasanguinis ↑<br>*Actinomyces odontolyticus* ↑
**Wu et al., 2012** [8]	China	CHB-LC, (n = 61),<br>LT (after LC) (n=74)<br>Healthy Control (n=38)	qPCR	***Lactobacillus* species**<br>*L. gasseri* ↑<br>*L. acidophilus* ↓<br>*L. reuteri*↓<br>*L. paracasei*↑<br>*L. crispatus* ↑<br>*L. rhamnosus* ↓<br>*L. fermentum* ↓<br>*L. salivarius* ↑
**Zeng et al., 2020** [9]	China	CHB (n =21),<br>CHB-LC (n = 25),<br>CHB-HCC (n =21),<br>Healthy Control (n = 15)	16S rRNA sequencing	**Phyla:**<br>*Actinobacteria* ↓<br>*Proteobacteria* ↑<br>*Bacteroidetes* ↑<br>*Firmicutes* ↓<br>**Genera:**<br>*Bifidobacterium* ↓
**Chen et al., 2020** [10]	China	HBV carriers (n=23)<br>CHB (n =28),<br>CHB-LC (n = 25),<br>Healthy Control (n = 21)	16S rRNA sequencing	**Phyla:**<br>*Actinobacteria* ↑<br>*Bacteroidetes* ↓<br>*Firmicutes* ↓<br>*Proteobacteria* ↑<br>**Genera:**<br>*Haemophilus* ↑<br>*Fusobacterium* ↑<br>*Veillonella* ↑<br>*Streptococcus* ↑

				Ruminococcus ↑<br>**Species**<br>*Dialister succinatiphilus* ↓<br>*Alistipes onderdonkii* ↓<br>*Barnesiella intestinihominis* ↓<br>*Bacteroides eggerthi* ↓<br>*Kineothrix alysoides* ↓<br>*Alistipes shahii* ↓
**Liu et al., 2019** [11]	China	CHB-HCC (n=35)<br>NBNC-HCC (n = 22)<br>Healthy Control (n=33)	16S rRNA sequencing	**Phyla:**<br>*Firmicutes* ↓<br>*Proteobacteria* ↓<br>**Genera:**<br>*Prevotella* ↑<br>*Lactobacillus* ↑<br>*Bifidobacterium* ↑<br>*Veillonella* ↑<br>*Ruminoclostridium* ↑<br>*Faecalibacterium* ↑<br>*Escherichia/Shigella*↑<br>*Buchnera* ↓<br>*Megamonas* ↓
**Wei et al., 2013** [12]	China	CHB-LC (n = 120):<br>Healthy Control (n = 120)	Illumina/Solexa sequencing and qPCR	**Phyla:**<br>*Bacteroidetes* ↓<br>*Proteobacteria* ↑<br>*Actinobacteria* ↑<br>**Genera:**<br>*Veillonella* ↑<br>*Bacteroides* ↓<br>*Clostridium* ↓

				**Species:**<br>*Veillonella Dispar* ↑<br>*Veillonella Parvula* ↑<br>*Escherichia Coli* ↑<br>*Klebsiella pneumonia* ↑<br>*Enterobacter cloaca* ↑<br>*Shigella dysenteriae* ↑<br>*Shigella flexneri* ↑<br>*Salmonella enteric* ↑<br>*Enterobacter cancerogenus* ↑<br>*Escherichia albertii*↑<br>*Bacteroides species* ↓
**Yang et al., 2020** [13]	China	HBV carriers (n=24)<br>CHB (n = 56)<br>CHB-LC (n = 54)<br>HBV-ACLF (n= 52)<br>Healthy controls (n=31)	16S rRNA sequencing	**Genera:**<br>*Bacteroides* ↓<br>*Roseburia* ↓<br>*Fusobacterium* ↓<br>*Enterococcus* ↑<br>*Escherichia–Shigella* ↑<br>*Streptococcus* ↑<br>*Klebsiella*↑
**Sun et al., 2021** [14]	China	CHB-NALT (n=92)<br>CHB-AALT (n=34)<br>Healthy controls (n=28)	16S rDNA sequencing	**Phyla:**<br>*Bacteroidetes* ↑<br>*Proteobacteria* ↑<br>**Genera:**<br>*Lactobacillus*↓<br>*Clostridium*↓<br>*Bifidobacterium*↓<br>**Species:**<br>*Lactobacillus salivarius*↓

				Bacteroides fragilis ↓
**Joo et al., 2021** [15]	South Korea	CHB (n=57)<br>Healthy controls (n=57)	16S rRNA sequencing	**Genera:**<br>*Alloprevotella*↑<br>*Paraprevotella*↑<br>*Hungatella*↑<br>*Mitsuokella*↑<br>**Species:**<br>*Bacteroides fragilis*↓<br>*Bacteroides coprocola*↑<br>*Bacteroides uniformis* ↑<br>*Eubacterium coprostanoligenes*↑
**Zheng et al., 2020** [16]	China	CHB (n=24)<br>LC (n=24)<br>HCC (n=75)<br>Healthy controls (n=20)	16S rRNA sequencing/ Illumina Hiseq	**Genera:**<br>*Neisseria* ↑<br>*Megamonas* ↑
**Huang et al., 2020** [17]	China	CHB-HCC (n=113)<br>Healthy controls (n=100)	16S rRNA sequencing/ MiSeq	**Genera:**<br>*Bacteroides*↑<br>*Lachnospiracea incertae sedis*↑<br>*Parabacteroides*↑<br>*Clostridium* ↑
**Shen et al., 2023** [18]	China	CHB (n=64)<br>Healthy controls (n=17)	16s rRNA sequencing	**Genera:**<br>*Fecalibacterium*↑<br>*Streptococcus*↑<br>*Sutterella* ↑<br>*Lachnospiraceae_ND-3007* ↑

				Turicibacter ↓<br>*Adlercreutzia*↓<br>*Ruminiclostridium9* ↑
**Zhang et al., 2023** [19]	China	CHB (n= 21),<br>LC (n= 25)<br>HCC (n= 21)<br>Healthy controls (n= 15)	16S rDNA sequencing	**Phyla:**<br>*Firmicutes* ↓<br>*Bacteroides* ↑<br>*Proteobacteria* ↑<br>**Genera:**<br>*Phascolarctobacterium*↓<br>*Akkermansia* ↑<br>*Barnesiella* ↑<br>*Bacteroides* ↑<br>*Blautia* ↓<br>*Fusicatenibacter* ↓<br>*Howardella* ↓<br>*Lachnospiraceae ND3007* ↓<br>*Marvinbryantia* ↓<br>*Butyricicoccus* ↓<br>*Ruminococcaceae CAG-352*↓<br>*Dialister*↓<br>*Eggerthella* ↓<br>*Ruminococcaceae UCG-013*↓<br>*Fluviicola*↑<br>*Veillonella*↑

				Cryomorphaceae__uncultured↑<br>*Flavobacteriaceae__uncultured* ↑<br>*Spongiibacteraceae BD1-7 clade*↑<br>*Lachnospiraceae UCG-008*↓<br>*Ruminococcaceae CAG-352*↓<br>*Ruminiclostridium 5* ↓<br>*Uncultured Erysipelotrichaceae* ↓
**Shu et al., 2022** [20]	China	CHB-LC (n=50)<br>Healthy controls (30)	16S rRNA Sequencing	**Phyla:**<br>*Firmicutes* ↓<br>*Bacteroides* ↑<br>***Genera:***<br>*Bifidobacterium*↓<br>*Lactobacillus*↓<br>*Steptococcus*↑<br>*Ruminococcus*↑<br>*Faecalibacterium* ↑<br>*Rothia*↑<br>*Blautia* ↑
**Yan et al., 2023** [21]	China	CHB-LC (n=30)<br>CHB-HCC (n=30)<br>Healthy controls (30)	16S rRNA Sequencing	**Phyla:**<br>*Firmicutes* ↓<br>*Bacteroides* ↓<br>*Proteobateria*↑<br>*Actinobaciota*↑<br>*Fusobasteriota*↑<br>**Genera:**<br>*Klebsiella*↑<br>*Streptococcus*↑<br>*Acinetobacter*↑<br>*Pantoea* ↑

				Proteus ↑<br>*Paenibacillus*↑<br>*Pseudomonas*↑<br>*Barnesiella* ↓<br>*Agathobacter*↓<br>*Ruminococcus*↓
**Li et al, 2022** [22]	China	CHB, Immune-tolerant (n=14)<br>CHB, Immune-active (n=10)<br>Healthy control (n=13)	16S rDNA sequencing	**IT-phase:**<br>**Phyla:**<br>*Bacteroidetes* ↑<br>**Genera:**<br>*Bacteroides* ↑<br>*Prevotella* ↑<br>*Megamonas*↑<br>*Senegalimassilia* ↑<br>*Alloprevotella* ↑<br>*Sutterella*↑<br>*Haemophilus*↑<br>**IA-phase:**<br>**Phyla:**<br>*Firmicutes*↑<br>**Genera:**<br>*Blautia* ↑<br>*Faecalibacterium*↑<br>*Clostridium innocuum group*↑<br>*Faecalitalea*↑
**Yao et al., 2021** [23]	China	HBV-ACLF (n=91)<br>Healthy control (n=30)	16S rDNA sequencing	**Phyla:**<br>*Bacteroidetes*↓<br>*Firmicutes*↑<br>*Proteobacteria*↑

				Actinobacteria↑<br>**Genera:**<br>*Veilonella*↑<br>*Streptococcus*↑<br>*Enterococcus*↑<br>*Klebsiella*↑<br>*Lactobacillus*↑<br>*Blautia*↑<br>*Prevotella*↓<br>*Megamonas*↓
**Li et al., 2022** [24]	China	CHB (n=23)<br>CHB-LC (n=20)<br>CHB-HCC (n=22)<br>Healthy controls (n=15)	16S rRNA Sequencing	**Genera:**<br>*Bilophila* ↓<br>*Lactobacillus* ↑<br>*Colidextribacter*↓<br>*Bilophila*↓<br>*Faecalibacterium*↓<br>*Monoglobus*↓<br>*Oscillibacter*↓<br>*Lachnospiraceae_ND3007_group*↓<br>*Eubacterium_ventriosum_group*↓
**Lin et al., 2023** [25]	Taiwan	Resolved HBV (n=14)<br>CHB (n=58)<br>CHB-LC (n=15)<br>CHB-HCC (n=19)<br>Healthy controls (n=56)	16S rRNA gene sequencing	**Phyla:**<br>*Firmicutes*↑<br>*Verrucomicrobiota*↑<br>*Fusobacteria* ↑<br>*Bacteroidetes* ↓<br>*Proteobacteria*↑<br>*Actinobacteriota*↑<br>**Genera:**<br>*Blautia*↑

			Veillonella↑<br>*Akkermansia* ↑<br>*Fusobacterium*↑<br>*Escherichia-Shigella*↑<br>*Streptococcus*↑<br>*Collinsella*↑

HBV: Hepatitis B virus infection; **CHB:** Chronic hepatitis B; **CHB-LC:** Chronic hepatitis B-related liver cirrhosis; **ALT:** Alanine aminotransferase; **LT:** Liver transplant; **HCC:** Hepatocellular carcinoma; **NBNC-HCC:** non-HBV non-HCV related hepatocellular carcinoma; **ACLF:** Acute-on-chronic liver failure; **CHB-NALT:** Chronic hepatitis B with normal alanine aminotransferase; **PCR-DGGE:** Nested-PCR-based denaturing gradient gel electrophoresis; **qPCR:** Quantitative polymerase chain reaction.↑: increase in abundance reported; ↓: decrease in abundance. Results shared with the present study in green; results discordant with the present study in red; and results not found in the present study in black.

References

1. Lu, H.; Wu, Z.; Xu, W.; Yang, J.; Chen, Y.; Li, L. Intestinal Microbiota Was Assessed in Cirrhotic Patients with Hepatitis B Virus Infection. Intestinal Microbiota of HBV Cirrhotic Patients. *Microb. Ecol.* **2011**, *61*, 693–703, doi:10.1007/s00248-010-9801-8.

2. Deng, Y.-D.; Peng, X.-B.; Zhao, R.-R.; Ma, C.-Q.; Li, J.-N.; Yao, L.-Q. The Intestinal Microbial Community Dissimilarity in Hepatitis B Virus-Related Liver Cirrhosis Patients with and without at Alcohol Consumption. *Gut Pathog.* **2019**, *11*, 58, doi:10.1186/s13099-019-0337-2.

3. Yun, Y.; Chang, Y.; Kim, H.-N.; Ryu, S.; Kwon, M.-J.; Cho, Y.K.; Kim, H.-L.; Cheong, H.S.; Joo, E.-J. Alterations of the Gut Microbiome in Chronic Hepatitis B Virus Infection Associated with Alanine Aminotransferase Level. *J. Clin. Med.* **2019**, *8*, E173, doi:10.3390/jcm8020173.

4. Xu, M.; Wang, B.; Fu, Y.; Chen, Y.; Yang, F.; Lu, H.; Chen, Y.; Xu, J.; Li, L. Changes of Fecal Bifidobacterium Species in Adult Patients with Hepatitis B Virus-Induced Chronic Liver Disease. *Microb. Ecol.* **2012**, *63*, 304–313, doi:10.1007/s00248-011-9925-5.

5. Wang, X.; Chen, L.; Wang, H.; Cai, W.; Xie, Q. Modulation of Bile Acid Profile by Gut Microbiota in Chronic Hepatitis B. *J. Cell. Mol. Med.* **2020**, *24*, 2573–2581, doi:10.1111/jcmm.14951.

6. Wang, J.; Wang, Y.; Zhang, X.; Liu, J.; Zhang, Q.; Zhao, Y.; Peng, J.; Feng, Q.; Dai, J.; Sun, S.; et al. Gut Microbial Dysbiosis Is Associated with Altered Hepatic Functions and Serum Metabolites in Chronic Hepatitis B Patients. *Front. Microbiol.* **2017**, *8*, 2222, doi:10.3389/fmicb.2017.02222.

7. Wang, K.; Zhang, Z.; Mo, Z.-S.; Yang, X.-H.; Lin, B.-L.; Peng, L.; Xu, Y.; Lei, C.-Y.; Zhuang, X.-D.; Lu, L.; et al. Gut Microbiota as Prognosis Markers for Patients with HBV-Related Acute-on-Chronic Liver Failure. *Gut Microbes* **2021**, *13*, 1921925, doi:10.1080/19490976.2021.1921925.

8. Wu, Z.-W.; Lu, H.-F.; Wu, J.; Zuo, J.; Chen, P.; Sheng, J.-F.; Zheng, S.-S.; Li, L.-J. Assessment of the Fecal Lactobacilli Population in Patients with Hepatitis B Virus-Related Decompensated Cirrhosis and Hepatitis B Cirrhosis Treated with Liver Transplant. *Microb. Ecol.* **2012**, *63*, 929–937, doi:10.1007/s00248-011-9945-1.

9. Zeng, Y.; Chen, S.; Fu, Y.; Wu, W.; Chen, T.; Chen, J.; Yang, B.; Ou, Q. Gut Microbiota Dysbiosis in Patients with Hepatitis B Virus-Induced Chronic Liver Disease Covering Chronic Hepatitis, Liver Cirrhosis and Hepatocellular Carcinoma. *J. Viral Hepat.* **2020**, *27*, 143–155, doi:10.1111/jvh.13216.

10. Chen, Z.; Xie, Y.; Zhou, F.; Zhang, B.; Wu, J.; Yang, L.; Xu, S.; Stedtfeld, R.; Chen, Q.; Liu, J.; et al. Featured Gut Microbiomes Associated With the Progression of Chronic Hepatitis B Disease. *Front. Microbiol.* **2020**, *11*, 383, doi:10.3389/fmicb.2020.00383.

11. Liu, Q.; Li, F.; Zhuang, Y.; Xu, J.; Wang, J.; Mao, X.; Zhang, Y.; Liu, X. Alteration in Gut Microbiota Associated with Hepatitis B and Non-Hepatitis Virus Related Hepatocellular Carcinoma. *Gut Pathog.* **2019**, *11*, 1, doi:10.1186/s13099-018-0281-6.

12. Wei, X.; Yan, X.; Zou, D.; Yang, Z.; Wang, X.; Liu, W.; Wang, S.; Li, X.; Han, J.; Huang, L.; et al. Abnormal Fecal Microbiota Community and Functions in Patients with Hepatitis B Liver Cirrhosis as Revealed by a Metagenomic Approach. *BMC Gastroenterol.* **2013**, *13*, 175, doi:10.1186/1471-230X-13-175.

13. Yang, X.-A.; Lv, F.; Wang, R.; Chang, Y.; Zhao, Y.; Cui, X.; Li, H.; Yang, S.; Li, S.; Zhao, X.; et al. Potential Role of Intestinal Microflora in Disease Progression among Patients with Different Stages of Hepatitis B. *Gut Pathog.* **2020**, *12*, 50, doi:10.1186/s13099-020-00391-4.

14. Sun, Z.; Huang, C.; Shi, Y.; Wang, R.; Fan, J.; Yu, Y.; Zhang, Z.; Zhu, K.; Li, M.; Ni, Q.; et al. Distinct Bile Acid Profiles in Patients With Chronic Hepatitis B Virus Infection Reveal Metabolic Interplay Between Host, Virus and Gut Microbiome. *Front. Med.* **2021**, *8*.

15. Joo, E.-J.; Cheong, H.S.; Kwon, M.-J.; Sohn, W.; Kim, H.-N.; Cho, Y.K. Relationship between Gut Microbiome Diversity and Hepatitis B Viral Load in Patients with Chronic Hepatitis B. *Gut Pathog.* **2021**, *13*, 65, doi:10.1186/s13099-021-00461-1.

16. Zheng, R.; Wang, G.; Pang, Z.; Ran, N.; Gu, Y.; Guan, X.; Yuan, Y.; Zuo, X.; Pan, H.; Zheng, J.; et al. Liver Cirrhosis Contributes to the Disorder of Gut Microbiota in Patients with Hepatocellular Carcinoma. *Cancer Med.* **2020**, *9*, 4232–4250, doi:10.1002/cam4.3045.

17. Huang, H.; Ren, Z.; Gao, X.; Hu, X.; Zhou, Y.; Jiang, J.; Lu, H.; Yin, S.; Ji, J.; Zhou, L.; et al. Integrated Analysis of Microbiome and Host Transcriptome Reveals Correlations between Gut Microbiota and Clinical Outcomes in HBV-Related Hepatocellular Carcinoma. *Genome Med.* **2020**, *12*, 102, doi:10.1186/s13073-020-00796-5.

18. Shen, Y.; Wu, S.-D.; Chen, Y.; Li, X.-Y.; Zhu, Q.; Nakayama, K.; Zhang, W.-Q.; Weng, C.-Z.; Zhang, J.; Wang, H.-K.; et al. Alterations in Gut Microbiome and Metabolomics in Chronic Hepatitis B Infection-Associated Liver Disease and Their Impact on Peripheral Immune Response. *Gut Microbes* **2023**, *15*, 2155018, doi:10.1080/19490976.2022.2155018.

19. Zhang, H.; Wu, J.; Liu, Y.; Zeng, Y.; Jiang, Z.; Yan, H.; Lin, J.; Zhou, W.; Ou, Q.; Ao, L. Identification Reproducible Microbiota Biomarkers for the Diagnosis of Cirrhosis and Hepatocellular Carcinoma. *AMB Express* **2023**, *13*, 35, doi:10.1186/s13568-023-01539-6.

20. Shu, W.; Shanjian, C.; Jinpiao, L.; Qishui, O. Gut Microbiota Dysbiosis in Patients with Hepatitis B Virus-Related Cirrhosis. *Ann. Hepatol.* **2022**, *27*, 100676, doi:10.1016/j.aohep.2022.100676.

21. Yan, F.; Zhang, Q.; Shi, K.; Zhang, Y.; Zhu, B.; Bi, Y.; Wang, X. Gut Microbiota Dysbiosis with Hepatitis B Virus Liver Disease and Association with Immune Response. *Front. Cell. Infect. Microbiol.* **2023**, *13*.

22. Li, Y.-N.; Kang, N.-L.; Jiang, J.-J.; Zhu, Y.-Y.; Liu, Y.-R.; Zeng, D.-W.; Wang, F. Gut Microbiota of Hepatitis B Virus-Infected Patients in the Immune-Tolerant and Immune-Active Phases and Their Implications in Metabolite Changes. *World J. Gastroenterol.* **2022**, *28*, 5188–5202, doi:10.3748/wjg.v28.i35.5188.

23. Yao, X.; Yu, H.; Fan, G.; Xiang, H.-P.; Long, L.; Xu, H.; Wu, Z.; Chen, M.; Xi, W.; Gao, Z.; et al. Impact of the Gut Microbiome on the Progression of Hepatitis B Virus Related Acute-on-Chronic Liver Failure. *Front. Cell. Infect. Microbiol.* **2021**, *11*.

24. Li, R.; Yi, X.; Yang, J.; Zhu, Z.; Wang, Y.; Liu, X.; Huang, X.; Wan, Y.; Fu, X.; Shu, W.; et al. Gut Microbiome Signatures in the Progression of Hepatitis B Virus-Induced Liver Disease. *Front. Microbiol.* **2022**, *13*.

25. Lin, M.-J.; Su, T.-H.; Chen, C.-C.; Wu, W.-K.; Hsu, S.-J.; Tseng, T.-C.; Liao, S.-H.; Hong, C.-M.; Yang, H.-C.; Liu, C.-J.; et al. Diversity and Composition of Gut Microbiota in Healthy Individuals and Patients at Different Stages of Hepatitis B Virus-Related Liver Disease. *Gut Pathog.* **2023**, *15*.
